# Enterohemorrhagic *Escherichia coli* O157 subclade 8b strains in Chiba Prefecture, Japan, produced larger amounts of Shiga toxin 2 than strains in subclade 8a and other clades

**DOI:** 10.1371/journal.pone.0191834

**Published:** 2018-01-30

**Authors:** Shinichiro Hirai, Eiji Yokoyama, Taku Wakui, Taichiro Ishige, Masaki Nakamura

**Affiliations:** 1 Division of Bacteriology, Chiba Prefectural Institute of Public Health, Chiba, Japan; 2 Division of Epidemiology, Chiba Prefectural Institute of Public Health, Chiba, Japan; 3 Genome Research Center, NODAI Research Institute, Tokyo University of Agriculture, Tokyo, Japan; USDA-ARS Eastern Regional Research Center, UNITED STATES

## Abstract

Enterohemorrhagic *Escherichia coli* O157 (O157) strains can be classified into clades (one of several phylogenetic groups) by single nucleotide polymorphisms (SNPs): these are clade 1, clade 2, clade 3, descendant and ancestral clades 4/5, clade 6, clade 7, clade 8, clade 9, and clade 12. Some recent studies showed that some O157 strains in clade 8 produced a larger amount of Shiga toxin (Stx) 2 than other strains. In this study, 1121 epidemiologically unlinked strains of O157 isolated in Chiba Prefecture, Japan were classified into clades during 1996–2014. Clade 8 strains were further classified into subclade 8a (67 strains) and subclade 8b (48 strains) using SNP analysis. In the absence of mitomycin C (MMC), subclade 8a strains in this study produced significantly greater amounts of Stx2 than subclade 8b strains. However, in the presence of MMC, the levels of Stx2 production in subclade 8b strains were significantly greater than subclade 8a strains. On the other hand, a recent study reported that the Stx2 production level in O157 strains was determined mainly by the subtypes of Stx2a phage (ϕStx2_α, β, γ, δ, ε, and ζ). Using O157 strains in this study, the Stx2a phages were classified into these subtypes. In this study, all strains of subclades 8a and 8b carried ϕStx2a_γ and ϕStx2a_δ, respectively. Some strains in clade 6 also carried ϕStx2a_δ. In the presence of MMC, subclade 8b strains produced significantly greater amounts of Stx2 than clade 6 strains carrying ϕStx2_δ. In this study, we propose that Stx2 production in subclade 8b strains in the presence of MMC might be enhanced due to genetic factors other than ϕStx2_δ.

## Introduction

Phylogenetic groups are groups of bacterial strains originating from a common ancestral clone [1, 2]. Since bacteria reproduce asexually, the strains in the same phylogenetic group possess similar or common genetic features e.g., biochemical properties [3], polymorphisms in the genome [4], and possession of pathogenic factors [5]. Recent studies have shown that enterohemorrhagic *Escherichia coli* O157 (O157) strains can be divided into several phylogenetic groups i.e., lineages [6], subgroups and clusters [7], and clades [8]. Yokoyama et al. [9] showed that the hierarchical relationship of the O157 phylogenetic groups was, in descending order, lineage, subgroup, cluster, and clade. In addition, Yokoyama et al. [9] suggested a paraphyletic model for O157 evolution based on these hierarchical relationships. This model has been further modified with new clade designations based on lineage analysis data (S1 Table) [10].

It has been reported that the pathogenicity of O157 strains varied between different clades. A higher percentage of clade 8 strains caused hemolytic uremic syndrome (HUS) than strains in other clades, suggesting strong pathogenicity of clade 8 strains [8, 11]. The strong pathogenicity of those O157 strains was due to Shiga toxin (Stx) 2 subtypes and the level of Stx2 production [12]. Stx2 were classified into several subtypes (i.e., Stx2a, Stx2b, Stx2c, Stx2d, Stx2e, Stx2f and Stx2g) [13]. Enterohemorrhagic *Escherichia coli* strains with Stx2a were more frequently isolated from HUS patients than the strains with the other Stx2 subtypes [14, 15]. Most clade 8 strains carry a *stx2a* gene and produce more Stx2 than strains in other clades [9, 16]. However, some O157 clade 8 strains produce less Stx2 than other clade 8 strains [17], indicating that clade 8 strains might be classified into two groups based on the difference in their Stx2 production level.

We have reported that clade 8 strains could be putatively classified into two groups based on the distribution of insertion sequence (IS) *629* in the O157 genome [18] i.e. the distributions of IS*629* in clade 8 strains were roughly divided into two different patterns. O157 strains in a phylogenetic group originated from a common ancestral clone. Therefore, the IS*629* distribution in O157 strains was similar in the same phylogenetic group. In contrast, the IS*629* distribution in the strains was markedly different between different phylogenetic groups. However, groups putatively classified by our study [18] could not be defined as phylogenetic groups. Since both IS*629* insertions and deletions occur in particular genome regions, the molecular trace of IS*629* insertions and deletions is not always left in the genome [19]. Therefore, the distribution of IS*629* is not useful as a marker for the definition of phylogenetic groups. A recent study showed that clade 8 could be classified into two phylogenetic groups (i.e., subclades 8a and 8b) based on single nucleotide polymorphisms (SNPs) [20].

That study also reported that O157 subclade 8a strains produced more Stx2 than strains in subclade 8b and other clades, since all subclade 8a strains carried a specific Stx2a phage subtype (i.e., ϕStx2a_γ). In a recent study [20], Stx2a phages are classified into six subtypes (α, β, γ, δ, ε, and ζ), and Stx2 production levels in O157 strains were mainly determined by the Stx2a phage subtypes. In particular, the levels of Stx2 production in O157 strains carrying ϕStx2a_γ were higher than those in strains carrying the other Stx2a subtypes [20]. Furthermore, the recent study showed that O157 strains in each clade and subclade carried particular subtypes of Stx2a phage (S2 Table) [20]. Although all subclade 8a strains and some clade 3 and clade 4/5 strains carried ϕStx2a_γ, the other strains with a *stx2a* gene had ϕStx2a_α, β, δ, ε, or ζ [20]. However, the level of Stx2 production was not the same among O157 strains carrying the same Stx2a phage subtype [20]. Therefore, although a small number of O157 strains were analyzed in a recent study [20], the difference in Stx2 production level in O157 strains needs to be evaluated using a large number of strains carrying different Stx2a phage subtypes.

In addition, O157 strains isolated over a long period of time should be analyzed to evaluate the difference in Stx2 production among strains in subclade 8a, subclade 8b, and other clades. However, in a recent study [20], strains in subclades 8a and 8b isolated in various areas of Japan in the 1990s were analyzed, although that study did not specify the detailed areas or years in which the strains were isolated. Strains of an O157 clone can often emerge and be disseminated in a particular area during a specific time period [21]. Most strains derived from an O157 clone have very similar genetic features [21, 22] e.g., Stx2 production, IS*629* distribution, and possession of pathogenic factors. For example, strains of a subclade 8a clone produced larger amounts of Stx2 than strains in all other subclade 8a clones, and strains derived from the subclade 8a clone emerged in other areas of Japan in the 1990s. Therefore, if O157 strains isolated in these areas in the 1990s were analyzed to evaluate Stx2 production levels in O157 clades, the levels in subclade 8a strains may have been biased.

Analysis of both Stx2 production in O157 strains and clinical symptoms in O157 patients is necessary to determine the pathogenicity of O157 phylogenetic groups. Several studies investigated the prevalence of the clades of O157 strains in patients with HUS and hemorrhagic colitis [8, 23], and suggested that clade 8 strains were the most pathogenic. However, in those studies, strains in subclades 8a and 8b were considered one phylogenetic group. Therefore, differences in the symptoms of O157 patients infected with strains in subclade 8a, subclade 8b and other clades needs to be re-investigated.

In this study, a large number of O157 strains isolated in Chiba Prefecture, Japan, (S1 Fig) during 1996–2014 were analyzed. These strains were classified into subclade 8a, subclade 8b, and other clades. Stx2a phages in these strains were classified into six subtypes. The level of Stx2 production was compared among strains in subclade 8a, subclade 8b and other clades. In addition, the prevalence of O157 strains in subclade 8a, subclade 8b and other clades in patients with bloody diarrhea were analyzed to determine the phylogenetic group with the strongest pathogenicity.

## Materials and methods

### O157 strains used in this study

In this study, epidemiologically linked O157 strains were multiple O157 strains isolated in an outbreak or from an intra-family infection. O157 infections in Japan are reported to local public health institutes according to the Act on Prevention of Infectious Diseases and Medical Care for Patients Suffering Infectious Diseases (Act No. 114 of 1998). Standard epidemiological studies are then conducted by these institutes. O157 infections are classified into sporadic cases, outbreak, or intra-family cases by standard epidemiological studies.

Epidemiologically unlinked O157 strains were selected as follows. When certain O157 strains are isolated from a patient, these strains can often be detected as one strain. We included all O157 strains isolated from sporadic cases. An O157 strain that was isolated first was selected from multiple O157 strains isolated in an outbreak or an intra-family scenario. A total of 1121 epidemiologically unlinked O157 strains were chosen from a collection of O157 strains isolated in 1996–2014 in Chiba Prefecture, Japan (S1 Fig).

These epidemiologically unlinked O157 strains were classified into clades defined by Manning et al. [8] using SNP analysis as previously described [24]. They were then classified into lineages by LSPA-6 [25] as previously reported [9]. Then, these epidemiologically unlinked strains were classified into clades revised by Hirai et al. [10] using data of both clades by Manning et al. [8] and lineages as previously reported [10] (S1 Table).

The distribution of IS*629* in the epidemiologically unlinked strains in each clade was investigated using IS-printing (Toyobo Co., Ltd., Osaka, Japan) [26]. The distribution of IS*629* among the strains in a clade was similar but the distribution was not the same. When the IS*629* distribution between two strains in a clade exhibited greater differences, the genetic features between these strains also displayed greater differences. In contrast, when the IS*629* distribution between two strains in a clade was more similar, genetic features between these strains were also correspondingly similar.

### Analyses of *stx*2 gene subtypes and Stx2 phages

Subtypes of the *stx2* gene were investigated as described by Wang et al. [27]. Stx2a phages were classified into six subtypes and the integration sites of the Stx2 phages were determined as described by Ogura et al. [20].

### Classification of clade 8 strains into subclades 8a and 8b

Since a SNP set for classifying clade 8 strains into subclades 8a and 8b was not available [20], a SNP set for this study was constructed as follows. Griffing et al. [28] developed a SNP set for epidemiological molecular subtyping of O157 strains using SNPs at 32 loci. This typing divided clade 8 strains into two subgroups. Several SNPs were selected from 32 SNPs used by Griffing et al. [28] to construct the SNP set for this study. To select these several SNPs, the SNPs at 32 loci used by Griffing et al. [28] were investigated using clade 8 strains with various genetic features (i.e., the various distribution of IS*629*). In this study, a minimum spanning tree (MST) was reconstructed from the distribution of IS*629* in the epidemiologically unlinked clade 8 strains (S2 Fig). A strain was selected from each of six widely-separated nodes in the MST as clade 8 strains with various genetic features.

Whole-genome sequence (WGS) analyses were conducted on these six strains, and the SNPs at the 32 loci used by Griffing et al. [28] were determined as previously described [29, 30]. Briefly, sequence libraries were prepared with Nextera XT DNA Sample Prep Kits (Illumina, Inc., San Diego, CA, USA), and 100 cycles of dual-index paired-end sequencing were carried out using the Illumina HiSeq2500 System (Illumina, Inc.). The Illumina analysis pipeline (CASAVA 1.6.0) was used for image analysis, base calling, and quality score calibration. Reads were sorted by barcode and exported to FASTQ files. Raw read data were deposited in the Sequence Read Archive in the DNA Data Bank of Japan (DDBJ, Bio Project No. PRJDB4016) (S3 Table). The FASTQ files were analyzed using CLC Genomics Workbench software, version 7.5 (CLC bio, Inc., Aarhus, Denmark). Read data were mapped to a reference genome (*E*. *coli* O157:H7 Sakai strain, GenBank Accession Number NC_002695) with the “Non-specific match was ignored” option. SNPs were detected with a fixed ploidy variant detection method with the “Coverage and count filters with Minimum coverage was 30” option for exclusion of ambiguous SNPs due to sequence reading errors. From the WGS analyses, 25 of the 32 SNPs were common to these six strains and 7 SNPs were uncommon: these 7 SNPs were ECs5052, 2440, 2538, 0418, 3540, 0580 and 2006 (S3 Table). In this study, clade 8 strains were divided into two subgroups using these 7 SNPs.

Six of the 7 SNPs that were not common in the six O157 clade 8 strains (i.e., ECs5052, 2440, 2538, 3540, 0580 and 2006) were analyzed by amplification refractory mutation system PCR (ARMS-PCR) [31]. In ARMS-PCR, two primer sets (i.e., one primer set consists of a forward and a reverse primer) are used to determine a SNP at a locus in the genome. A PCR is carried out twice using each of two primer sets. Since one of two alleles at a SNP locus is amplified by a PCR using either of two primer sets, the SNP at the locus is determined. The primers used for ARMS-PCR in this study are listed in S4 Table. Amplification for ARMS-PCR was done by touchdown PCR using FastStart Taq DNA Polymerase (Roche Co., Ltd., Basel, Switzerland). Briefly, the first cycle was at 95°C for 5 min; followed by 5 cycles at 95°C for 30 s, annealing at Tm+5°C for 30 s (with the annealing temperature decreasing by 1°C for each cycle). After the 5 cycles, 33 or 35 cycles were performed with 95°C for 30 s, annealing at Tm for 30 s, and 72°C for 30 s; then 1 cycle at 72°C for 7 min was performed. After PCR amplification, the SNP in each locus was determined by agarose gel electrophoresis.

The 7th SNP (i.e., ECs0418) in the SNP set in this study could not be determined using ARMS-PCR. PCR amplicons for ECs0418 were observed by ARMS-PCRs using both of two primer sets, or they were not observed. Although ARMS-PCR primers for ECs0418 were improved, the 7th SNP could not be determined. Therefore, the 7th SNP was analyzed by Sanger DNA sequencing. Sanger DNA sequencing consisted of five steps i.e., PCR, purification of PCR amplicons, sequencing reactions, removal of excess dyes, and DNA sequence analysis. Amplification for PCR was done using TaKaRa Taq Hot Start Version (Takara Bio, Inc., Shiga, Japan). The primers used for this PCR are listed in S5 Table. The first PCR cycle was at 95°C for 5 min; followed by 30 cycles at 95°C for 30 s, annealing at 60°C for 30 s, and 72°C for 1 min, and then 1 cycle at 72°C for 7 min. After PCR amplification, the amplicons were purified using a QIAamp DNA Micro Kit (Qiagen Co., Ltd., Venlo, Netherlands). The sequencing reactions for the purified amplicons were carried out using a BigDye Terminator v3.1 Cycle Sequencing Kit (Thermo Fisher Scientific, Inc., Waltham, MA, USA) and the primers listed in S5 Table. The first sequencing reaction cycle was at 95°C for 5 min; followed by 25 cycles at 96°C for 10 s, annealing at 50°C for 5 s, and 60°C for 4 min. After each sequencing reaction, excess terminators were removed using a BigDye XTerminator Purification Kit (Thermo Fisher Scientific, Inc.). Finally, the sequencing reaction products were analyzed using a Genetic Analyzer 310 capillary gel electrophoresis sequencer (Applied Biosystems, CA, USA).

The epidemiologically unlinked O157 clade 8 strains were classified into subclades 8a and 8b as follows. Maximum likelihood phylogenetic tree (MLPT) analysis was carried out using Molecular Evolutionary Genetics Analysis (MEGA) software, version 6.0. [32]. ARMS-PCR and Sanger DNA sequencing data for the 7 SNPs was imported into the software and an MLPT was reconstructed using the nearest-neighbor interchange method and the Tamura-Nei model. The result was the separation of the clade 8 strains into two subgroups in the MLPT. The MLPT subgroups were determined to be subclade 8a and subclade 8b by analyses of the *stx2* gene subtypes [27], Stx2a phage subtypes [20] and Stx2 phage integration sites [20].

### Population genetics analyses of subclade 8a and 8b strains

The difference in genetic diversity between O157 strains in subclades 8a and 8b was evaluated using *Φ*_*PT*_, which is an analogue of *Fst* [33], from IS*629* distribution data. The Genetic Analysis in Excel (GenAlEx), version 6.5 software, an add-in package of Microsoft Excel, was used to calculate the *Φ*_*PT*_ value with 999 permutations. *Φ*_*PT*_ was calculated as the proportion of the variance among populations from:

$$\phi_{PT}=\frac{V_{AP}}{V_{AP}+V_{WP}}$$

where *V*_A*P*_ was the variance among populations and *V*_*WP*_ was the variance within a population. A *Φ*_*PT*_ value significantly different from zero indicated a genetic difference between subclade 8a and 8b strains. Therefore, a *Φ*_*PT*_ value significantly different from zero for the subclades 8a and 8b strains in this study would mean that their classification into these two groups using the data for 7 SNPs (S3 Table) was accurate. However, if clade 8 strains were not accurately classified into subclades 8a and 8b, the difference in diversity of the strains in these two populations would be small and a significant *Φ*_*PT*_ value would be not generated.

The genetic diversity of O157 strains in subclade 8a and subclade 8b was evaluated by the Hunter Gaston discriminatory index (HGDI), determined from multilocus variable-number tandem repeat analysis (MLVA) [34, 35]. HGDI indicates the discriminatory power of the typing method used for the bacterial strains. HGDI was calculated from the number and frequencies of these types. An HDGI of 1.0 indicates that a typing method was able to distinguish each strain in a population from all other strains in that population. In other words, when a typing method is used to type the strains in a population, an HGDI of 1.0 indicates that the diversity of the population is infinite. In this study, subclade 8a and subclade 8b strains were analyzed by MLVA as described by Izumiya et al. [35], and these strains were classified into MLVA types. The HGDI for the O157 strains in each subclade was calculated from:

D=1−1N(N−1)∑j=1Snj(n−j1)

where *D* is the HGDI, *N* is the total number of strains in each subclade, *S* is the total number of MLVA types, and *n*_*j*_ is the number of strains belonging to the *j*_*th*_ MLVA type.

### Comparison of Stx2 production among strains in subclade 8a, subclade 8b and other clades

Stx2 production was measured using all of the epidemiologically unlinked strains in subclades 8a and 8b in this study. In addition, Stx2 production was measured for several epidemiologically unlinked strains selected from clades other than subclades 8a and 8b. To select these strains, the IS*629* insertion distribution data for strains in each clade was imported into BioNumerics, version 5.0 software (Applied Maths, Sint-Martenes-Latem, Belgium). These IS*629* insertion distribution data was used to reconstruct a MST for strains in each clade by calculation of a simple matching coefficient and a binary coefficient (S3 Fig). Several strains in each clade were selected from widely-separated nodes in the MST for Stx2 production assays. Since these strains in each clade can have different genetic features, the mean level of Stx2 production in these strains should approximate the mean level for all strains in that clade.

The Stx2 production levels of O157 strains were measured as follows. Each strain was inoculated into 3 ml CAYE medium (Denka Seiken Co. Ltd., Niigata, Japan) at 37°C with shaking and grown to mid-log phase. Mitomycin C (MMC) (Sigma-Aldrich Co., Ltd., USA) then was added to each culture to a final concentration of 0.5 μg/ml. After an additional 3 h incubation, each culture was then centrifuged (7,700 ×*g* for 10 min) and its supernatant was assayed for Stx2. MMC-untreated O157 strains were prepared similarly but without the addition of MMC. The supernatants were serially diluted 2-fold using the dilution buffer in the VTEC-Reversed Passive Latex Agglutination (VTEC-RPLA) assay kit (Denka Seiken Co. Ltd., Niigata, Japan). The Stx2 titer of each O157 strain was determined using the VTEC-RPLA assay kit following the manufacturer’s instructions. The Stx2 titers were assayed for strains in subclade 8a, subclade 8b and other clades.

### Analysis of the prevalence of strains in subclade 8a, subclade 8b and other clades in patients with bloody diarrhea

Epidemiologically unlinked O157 strains were linked to clinical information of patients infected with these strains as follows. In 2006, the National Epidemiological Surveillance of Infectious Diseases (NESID) database was constructed by the Japanese Government to survey infectious diseases in Japan. O157 infections in Japan are reported to local public health institutes according to Act No. 114 of 1998. These institutes compile the personal and clinical information of O157 patients and input this information into a database. In addition, O157 strains isolated from patients in Chiba Prefecture are sent to the Chiba Prefectural Institute of Public Health with documentation including patient names (S1 Fig). In this study, epidemiologically unlinked O157 strains isolated in 2006–2014 in Chiba Prefecture were linked to personal and clinical information of patients using NESID and other documentation. Since NESID was not constructed until 2005, symptom information for O157 patients in 1996–2005 was unavailable. After the patient and O157 strain data were linked, all personal information from the linked data was deleted to anonymize the linked data in this study.

The prevalence of O157 strains in subclade 8a, subclade 8b and other clades in patients with various clinical symptoms was determined in this study using the anonymized information (S6 Table). Patients with bloody diarrhea were selected from patients with various symptoms to determine the phylogenetic group with the strongest pathogenicity. The prevalence of strains in subclade 8a, subclade 8b and other clades in patients with bloody diarrhea was analyzed. The diagnostic criterion for bloody diarrhea was as follows. A doctor visually observed a stool of an O157 patient. When the stool included fresh blood and was watery, the patient was diagnosed as having bloody diarrhea.

### Statistical analysis

Statistical analyses were done using the Statcel3 software (OMS Inc. Saitama, Japan) add-on package for Microsoft Excel. P < 0.05 was considered to be significant for all analyses. The Stx2 production levels were compared from O157 strains in subclade 8a, subclade 8b and other clades using the Kruskal-Wallis test. If a significant difference was observed, a pairwise comparison of the strains in two of the subclades and/or clades was carried out using the Mann-Whitney *U* test. The prevalence of O157 patients with bloody diarrhea was compared for strains in subclade 8a, subclade 8b and other clades using the Chi-square test. If a significant difference was observed, a pairwise comparison of strains in the two subclades and/or clades was carried out using Fisher′s exact test.

### Ethics statement

This study did not involve human participants, human tissue, vertebrate animals, or vertebrate animal embryos and tissues. Therefore, this study was not conducted on either humans or animals.

Analysis of O157 patient clinical data in this study was approved by the Committee on the Ethics of Chiba Prefectural Institute of Public Health (Permit Number: 45). In this study, personal information from O157 patients was collected and anonymized using the methods approved by the Ethics Committee. Then, clinical data from the patients was analyzed using the anonymized information. Therefore, the analysis of clinical data in this study was conducted without breaching patient confidentiality.

## Results

### Classification of clade 8 strains into subclades 8a and 8b

SNP and LSPA-6 analyses classified 121 of the epidemiologically unlinked O157 strains in this study as clade 8 (S7 Table). Two of the clade 8 strains did not carry a *stx2a* or *stx2c* gene. This result contradicted the O157 evolutionary model of Yokoyama et al. [9] and, therefore, these two strains were excluded from this study.

**Fig 1:**
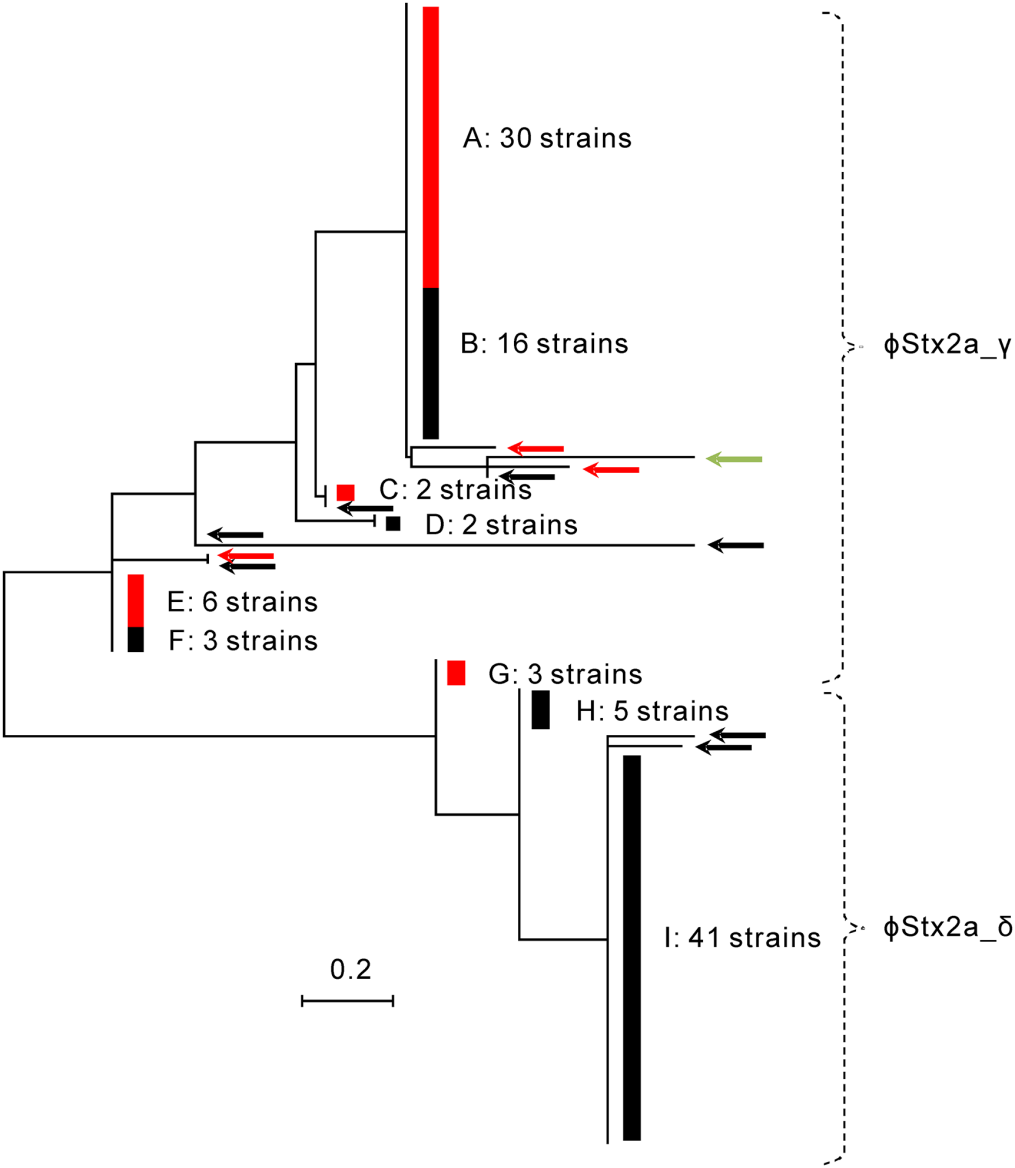
MLPT reconstructed from SNP data of clade 8 strains analyzed using the SNP set in this study. The dashed line marks branches with Stx2a phage subtypes. Red bars (A, C, E and G) indicate branches with multiple strains with both *stx2a* and *stx2c* genes. Red arrows indicate strains with both *stx2a* and *stx2c* genes. Black bars (B, D, F, H and I) indicate branches with multiple strains with only *stx2a*. Black arrows indicate strains with *stx*2a. The green arrow indicates a strain with a Stx2a phage that could not be typed.

An MLPT reconstructed using data for 7 SNPs showed that clade 8 strains were divided into two subgroups (Fig 1 and S3 Table). All of the clade 8 strains in the first subgroup carried a *stx2a* gene or both *stx2a* and *stx2c* genes (S8 Table). The Stx2a and Stx2c phages in these strains were integrated into the *argW* and *sbcB* loci in the O157 genome, respectively. Although one clade 8 strain in this subgroup carried a Stx2a phage that could not be typed, the other strains carried ϕStx2a_γ. Ogura et al [20] showed that subclade 8a strains carried an *stx2a* gene or both *stx2a* and *stx2c* genes, and that Stx2a and Stx2c phages in these strains were integrated in the *argW* and *sbcB* loci in the O157 genome, respectively (S2 Table). In their report [20], the Stx2a phages in subclade 8a strains were classified as ϕStx2a_γ. Based on the properties of O157 subclades 8a as defined by the recent study of Ogura et al. [20], the clade 8 strains in the first subgroup were designated subclade 8a strains. The strain with a Stx2a phage that could not be typed was excluded from this study: this strain is indicated by a green arrow in Fig 1. Therefore, 67 of the 121 clade 8 strains in this study were classified as subclade 8a strains.

Most of the clade 8 strains in the second subgroup in the MLPT carried an *stx2a* gene (Fig 1 and S8 Table). The Stx2a phages in these strains were integrated into the *argW* locus in the O157 genome. These phages were classified as ϕStx2a_δ. Ogura et al [20] reported that subclade 8b strains carried a *stx2a* gene, and that the Stx2a phages in subclade 8b strains were integrated in the *argW* locus (S2 Table). In their report [20], the Stx2a phages in these strains were classified as ϕStx2a_δ. Based on the properties of O157 subclades 8b as defined by a recent study [20], most of the clade 8 strains in the second subgroup were designated subclade 8b strains.

However, three strains in the second subgroup carried both *stx2a* and *stx2c* genes: these are the G strains in Fig 1. The Stx2a and Stx2c phages in the G strains were integrated into the *argW* and *sbcB* loci in the O157 genome, respectively (S8 Table). The Stx2a phages in the G strains were classified as ϕStx2a_γ. Although G strains had the property of subclade 8a strains as defined by the aforementioned study of Ogura et al [20] (S2 Table), these strains clustered with subclade 8b strains using the SNP set for this study. Since it could not be determined that G strains were ether subclade 8a or 8b strains, G strains were excluded from this study. Therefore, 48 of the 121 clade 8 strains in this study were classified as subclade 8b strains.

### Population genetics analyses of strains in subclades 8a and 8b

Population genetics analyses were carried out to evaluate the genetic diversity of the strains in subclades 8a and 8b that were isolated in Chiba Prefecture in 1996–2014 (S1 Fig). The *Φ*_*PT*_ value between the strains in subclades 8a and 8b calculated from the IS*629* distribution data was 0.508 (p < 0.001), indicating a significant genetic difference between subclade 8a and 8b strains. The HGDIs calculated from the MLVA data for strains in subclades 8a and 8b were 0.9891 and 0.9699, respectively, indicating that the genetic diversity of these subclade 8a and 8b strains was high.

### Stx2 production levels among strains in subclade 8a, subclade 8b and other clades

**Fig 2:**
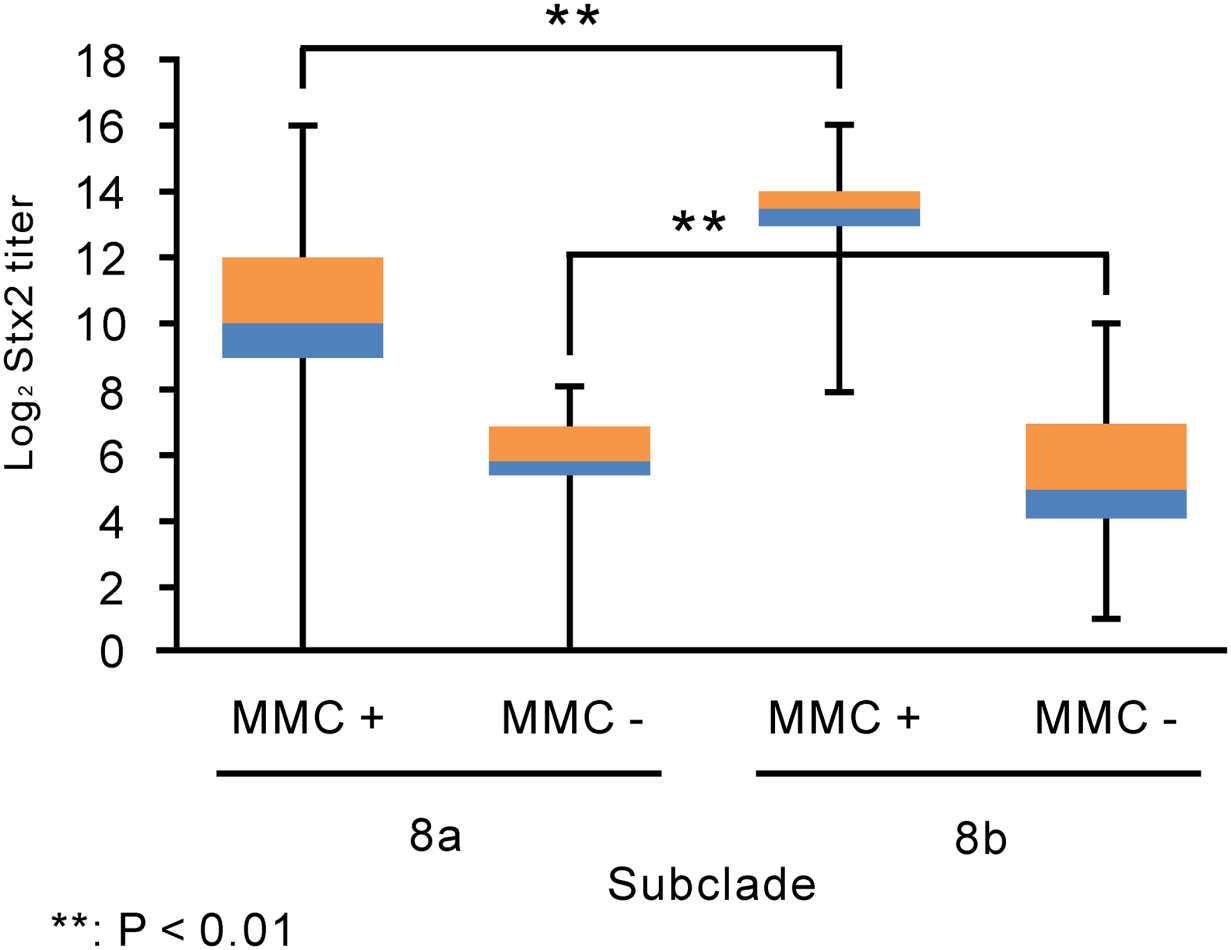
The levels of Stx2 production in subclade 8a and 8b strains. The Stx2 titers of the subclade 8a and 8b strains in this study in the presence and absence of MCC are shown using the box-and-whisker plot. The bottoms of the lower bars show the minimum of Stx2 titers. The blue boxes show the Stx2 titers from the 25th percentile to the median. The orange boxes show the Stx2 titers from the median to the 75th percentile. The tops of the upper bars show the maximum of Stx2 titers. MMC + and MMC—indicate O157 strains that were treated and non-treated with MMC, respectively.

The level of Stx2 production in the presence and absence of MMC was compared between subclade 8a and 8b strains (Fig 2, S4 Fig and S8 Table). In the absence of MMC, Stx2 production was significantly higher in subclade 8a strains than in subclade 8b strains (P < 0.01); the median Stx2 titers in subclade 8a and 8b strains were 64 and 32, respectively. In the presence of MMC, Stx2 production was significantly lower in subclade 8a strains than subclade 8b strains (P < 0.01); the median Stx2 titers in subclade 8a and 8b strains were 1024 and 11585, respectively.

**Fig 3:**
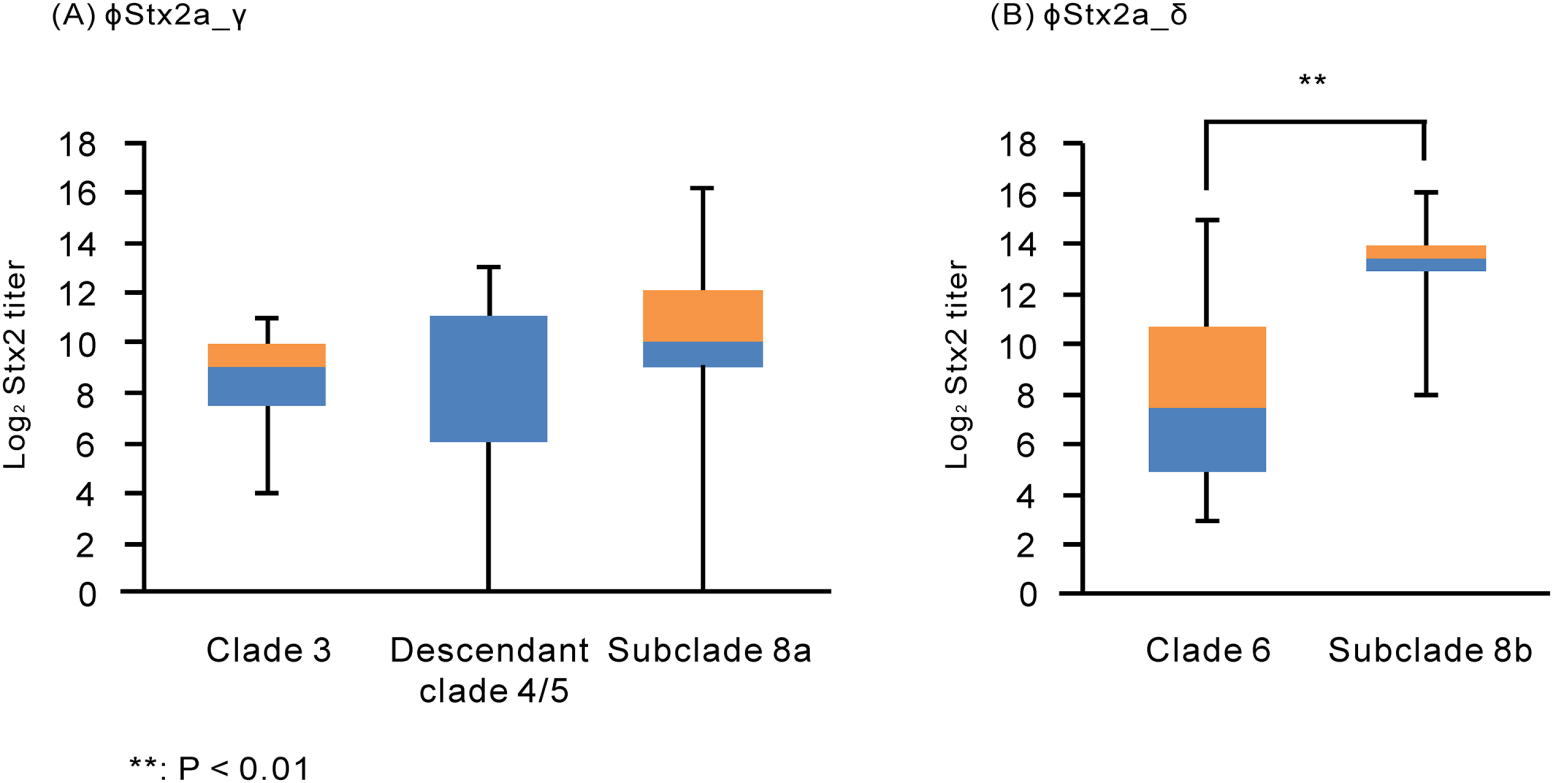
The levels of Stx2 production in O157 strains carrying ϕStx2a_γ or ϕStx2a_δ with MMC treatment. The Stx2 titers in O157 strains carrying (A) ϕStx2a_γ or (B) ϕStx2a_δ in the presence of MCC are shown using the box-and-whisker plot. The bottoms of the lower bars show the minimum of Stx2 titers. The blue boxes show the Stx2 titers from the 25th percentile to the median. The orange boxes show the Stx2 titers from the median to the 75th percentile. The tops of the upper bars show the maximum of Stx2 titers.

Stx2 production of O157 strains carrying ϕStx2a_γ in the presence of MCC was compared between strains in subclade 8a, clade 3 and descendant clade 4/5 (Fig 3A and S8 and S9 Tables). Eight of 21 clade 3 strains and 7 of 10 descendant clade 4/5 strains carried ϕStx2a_γ (S9 Table). In the presence of MMC, Stx2 production of strains carrying ϕStx2a_γ was not significantly different among subclade 8a, clade 3 and descendant clade 4/5 strains (P ≥ 0.05). In addition, in the presence of MMC, Stx2 production of strains carrying ϕStx2a_δ was compared between strains in subclade 8b and clade 6 (Fig 3B and S8 and S9 Tables). Ten of 13 strains in clade 6 carried ϕStx2a_δ (S9 Table). In the presence of MMC, Stx2 production in subclade 8b strains was significantly higher than in clade 6 strains with ϕStx2a_δ (P < 0.01).

### Prevalence of O157 patients with bloody diarrhea that are infected with strains in subclade 8a, subclade 8b and other clades

O157 patients infected with strains in clade 1, descendant and ancestral clade 4/5, clade 6 and clade 9 were excluded from this analysis because of the small number of patients with bloody diarrhea infected with the strains in these clades: 0 strains in clade 1, 8 strains in descendant clade 4/5, 0 strains in ancestral clade 4/5, 9 strains in clade 6 and 0 strains in clade 9. The prevalence of O157 patients with bloody diarrhea infected with subclade 8a strains was significantly higher than for clade 2, clade 7 and clade 12 strains (P < 0.05), although the prevalence was not significantly higher than for subclade 8b and clade 3 strains (Table 1). The prevalence of O157 patients with bloody diarrhea infected with a clade 7 or clade 12 strain was significantly lower than for strains in clades other than clades 7 and 12 (P < 0.05). On the other hand, analysis of the prevalence of strains in clades and subclades in patients with HUS is useful to determine the phylogenetic group with the strongest pathogenicity [8]. However, the analysis could not be conducted due to the small number of patients with HUS in this study (S6 Table).

**Table 1:** Pairwise comparison of the number of strains from patients with and without bloody diarrhea between two clades ^a^.

**Clade**	**2**	**3**	**7**	**Sub 8a** ^b^	**Sub 8b** ^c^	**12**	**Number of strains**
**2**		ns	1.62	0.52	ns	2.67	137
**3**	0.2973		1.89	ns	ns	3.11	158
**7**	0.0393	0.0075		0.32	0.38	ns	111
**Sub 8a** ^b^	0.0387	0.0918	0.0009		ns	5.11	53
**Sub 8b** ^c^	0.2016	0.3100	0.0283	0.4618		4.29	25
**12**	0.0022	0.0003	0.1014	0.0000	0.0034		59

^a^ Values above the diagonal line are odds ratio values. ns, not significant. Values under the diagonal line are P values calculated by Fisher’s exact test (P < 0.05).

^b^ Subclade 8a.

^c^ Subclade 8b.

## Discussion

A recent study by Ogura et al. [20] reported that O157 clade 8 strains were classified into subclades 8a and 8b, and that subclade 8a strains produced larger amounts of Stx2 than strains in subclade 8b and other clades. However, the results of our study reported here were not in agreement with that of a recent study [20]. In our study, subclade 8b strains produced significantly more Stx2 in the presence of MMC than subclade 8a strains (Fig 2, S4 Fig and S8 Table). This difference may be due to the difference in the number of strains analyzed in the two studies. In this study, we compared Stx2 production between subclade 8a and 8b strains using a large number of O157 strains isolated in Chiba Prefecture during 1996–2014 (S1 Fig). In contrast, a few subclade 8a and 8b strains that had been isolated in various areas of Japan in the 1990s were analyzed by Ogura et al [20].

In this study, clade 8 strains were correctly classified into subclade 8a and 8b using a defined set of 7 SNPs (S3 Table). The SNP set in our study was different from a SNP set used by Ogura et al. [20]. If clade 8 strains in this study were incorrectly classified into subclade 8a and 8b, the results of this study would be different from that of the aforementioned study [20]. The *Φ*_*PT*_ value calculated from the IS*629* distribution data of the subclade 8a and 8b strains was significantly different than zero, indicating that strains in these two subgroups were correctly divided into phylogenetically different groups. Furthermore, analysis of the *stx2* genes and Stx2a phages in the strains in the two subgroups in our study confirmed that they were subclade 8a and 8b strains, respectively (S8 Table). The recent study by Ogura et al. [20] reported that subclade 8a strains carried a *stx2a* gene or both *stx2a* and *stx2c* genes, and that the Stx2a and Stx2c phages were integrated into the *argW* and *sbcB* loci, respectively (S2 Table). The Stx2a phages in subclade 8a strains were classified as ϕStx2a_γ in the study by Ogura et al. [20]. In their report [20], subclade 8b strains carried a *stx2a* gene, and the Stx2a phages in subclade 8b strains were integrated in the *argW* locus and were classified as ϕStx2a_δ.

Although G strains clustered with subclade 8b strains using the SNP set for this study (Fig 1), these strains had the property of subclade 8a strains as defined by a recent study [20] (S2 and S8 Tables). Therefore, G strains were excluded from this study. However, the Stx2a phages in subclades 8a and 8b strains were not investigated by researchers other than Ogura et al. [20], and thus, further research is needed. It is possible that G strains might be classified into subclade 8b. However, the ratio of three G strains for all subclade 8b strains used in this study was low. Even if three G strains were not excluded from this study, the level of Stx2 production in subclade 8a strains was significantly smaller than the levels produced by subclade 8b strains in the presence of MMC.

The levels of Stx2 production in O157 strains by the assays used in this study were correlated with those by the assays used in the recent study of Ogura et al. [20] (S5 Fig). In that recent study [20], after O157 strains were cultured in CAYE medium, the cells were lysed using polymyxin B to release intracellular Stx2. Then, to measure total Stx2 production, Stx2 in both the cell lysates and medium supernatants was measured. In our study, Stx2 was measured only in the medium supernatants, without polymixin B treatment. Stx2 is produced in lysogenized O157 cells after Stx2a phage transcription begins in the O157 genome [36]. Subsequent prophage induction and lytic growth leads to release of the intracellular Stx2. If the cells are not lysed, Stx2 remains in the cells after it is produced. For O157 cells in CAYE medium, if the majority of cells retain intracellular Stx2, there may be a significant difference between the Stx2 measured in this study and that measured by Ogura et al. [20]. Pearson’s correlation coefficient was calculated for the level of Stx2 production measured by the method used for the subclade 8a and 8b strains in this study (S5 Fig) and that used for the strains in the study of Ogura et al. [20]. Pearson’s correlation coefficient was *r* = 0.946, indicating that these methods had strong positive correlation.

Epidemiologically unlinked O157 strains were selected for this study so we could analyze genetically diverse strains in subclades 8a and 8b. The genetic diversity of strains derived from a clone is very low [21], and such strains show very similar levels of Stx2 production. If strains derived from a clone were included in the analysis of subclade 8a and 8b strains, the Stx2 production levels obtained for subclade 8a and 8b strains would be biased. The HGDIs of the subclade 8a and 8b strains in our study were 0.9891 and 0.9699, respectively, indicating that the genetic diversity of these subclade 8a and 8b strains was high. This high genetic diversity indicated that either strains derived from a clone were not included in the analysis of subclade 8a and 8b strains in this study or that only a few strains derived from a clone were included in this study. We also investigated whether subclade 8a or 8b strains emerged from clones during 1996–2014 in Chiba Prefecture (S1 Fig). When the strains in subclades 8a and 8b, that were isolated in 1996–2014, were divided into 4 time periods and analyzed (i.e., 1996–2000, 2001–2005, 2006–2010 and 2011–2014), there was no significant difference in the *Φ*_*PT*_ values for the strains in these periods. If subclade 8a or 8b strains had emerged from a clone during one of these time periods, the genetic diversity of the strains in that subclade would have decreased during that period, leading to a significant difference in the calculated *Φ*_*PT*_ value [24].

Although two strains of all strains in subclade 8a analyzed for this study did not produce significant levels of Stx2 (Fig 2, S4 Fig and S8 Table), the difference in the results of this study and the study by Ogura et al. [20] could not be attributed to these two strains. For example, when the Stx2 phage in the O157 genome was mutated, the O157 strain could not produce Stx2 or did not produce significant levels of Stx2. The mutated strain was atypical, and should be excluded from comparisons of the levels of Stx2 production in strains among phylogenetic groups. Therefore, two subclade 8a strains with < 1 of Stx2 titers in the absence and presence of MMC might be atypical in this study (Fig 2, S4 Fig and S8 Table). However, the ratio of two strains for all strains in subclade 8a was low in this study. Even if these two strains were excluded from subclade 8a strains analyzed by this study, the levels of Stx2 production in subclade 8a strains were significantly smaller than the levels in subclade 8b strains in the presence of MMC.

This study suggested that genetic factors in addition to the Stx2a phage subtype could enhance Stx2 production in subclade 8b strains in Chiba Prefecture. A recent study reported that the Stx2 production level in O157 strains was mainly determined by the Stx2a phage subtype [20]. In addition, that study showed that the Stx2 level in strains carrying ϕStx2a_γ was higher than in strains carrying other phage subtypes [20]. In both our study and the recent study [20], strains in subclades 8a and 8b carried ϕStx2a_γ and ϕStx2a_δ (Fig 1 and S8 Table), respectively. In our study, the level of Stx2 production in subclade 8a strains was significantly higher than in subclade 8b strains in the absence of MMC (Fig 2, S4 Fig and S8 Table), in agreement with the recent study [20]. However, our study showed that the level of Stx2 production in subclade 8b strains was significantly higher in the presence of MMC than in subclade 8a strains (Fig 2, S4 Fig and S8 Table) and in clade 6 strains carrying ϕStx2a_δ (P < 0.01) (Fig 3B and S9 Table).

Recent studies reported that some genetic factors, other than the Stx2a phage subtype, enhanced the level of Stx2 production in O157 strains. Stx2 production in O157 strains was reported to be enhanced by deletion of anaerobic nitric oxide reductase genes (*norV*) [37, 38]. A recent study suggested that the level of Stx2 production in O157 strains was affected by insertion of IS*629* into the Stx2a phage genome and by the presence of SNPs in the phage genome [39]. Some studies showed that O157 strains carrying two Stx2 phages in the genome produced a smaller amount of Stx2 than those carrying only one Stx2 phage [40, 41]. These studies suggested that lower Stx2 production in the strains with two Stx2 phages might be due to regulation by CI repressors of both phages operating in *trans* [40, 41]. In the presence of MMC, the subclade 8b strains isolated in Chiba Prefecture may increase Stx2 production by any of these genetic factors e.g., deletion of *norV*, insertion of IS*629*, presence of SNPs and possession of more than one Stx2 phage.

As described above, the genetic factors for enhancement of Stx2 production also may be involved in the different levels of Stx2 production in subclade 8b strains in this study and in a recent study [20]. The study of Ogura et al. [20] evaluated Stx2 production in subclade 8b strains isolated in various areas of Japan in the 1990s. In our study, Stx2 production was analyzed in subclade 8b strains isolated in Chiba Prefecture in 1996–2014 (S1 Fig). However, the subclade 8b strains in Japan in the 1990s might not yet have acquired the genetic factors for enhancement of Stx2 production. In general, the O157 strains in the same phylogenetic group possess similar or common genetic features [2, 3, 5]. However, the genetic factors would be hindered by classification of O157 strains into clades and subclades. To identify such genetic factors, a WGS analysis should be carried out using the subclade 8b strains in this study and in the recent study [20].

Although subclade 8b strains produced significantly more Stx2 in the presence of MMC than subclade 8a strains (Fig 2, S4 Fig and S8 Table), the prevalence of O157 patients with bloody diarrhea was not significantly different between patients with subclade 8a and 8b strains (Table 1). The O157 strains analyzed in this study were isolated from symptomatic patients and asymptomatic carriers. The asymptomatic carriers were identified by fecal examinations of a food supply worker and of a person who had contact with O157 patients. However, other asymptomatic carriers in Chiba Prefecture were not identified by these investigations (S1 Fig). The asymptomatic carriers identified in this study were only a few of the possible asymptomatic carriers in Chiba Prefecture. Therefore, the strong pathogenicity of subclade 8b strains identified in this study may not be correct due to the unknown number of unidentified asymptomatic carriers. However, recent studies have suggested that overexpression of proteins other than Stx2 may be involved in the strong pathogenicity of some O157 strains [42, 43] e.g. the curli production protein CsgC, a transcriptional activator PchE, and a serine protease autotransporter enterotoxin EspP. It is not known whether the expression levels of such proteins in subclade 8b strains may or may not be higher than in strains in subclade 8a or other clades.

In conclusion, subclade 8b strains isolated in Chiba Prefecture produced more Stx2 than strains in subclade 8a and other clades in the presence of MMC. Although these subclade 8b strains were isolated in Chiba Prefecture during 1996–2014, they may not have been prevalent in other areas of Japan in the 1990s. The level of Stx2 production in subclade 8b strains isolated in Chiba Prefecture in the presence of MMC may be enhanced by genetic factors other than ϕStx2_δ. To identify these genetic factors, a WGS analysis should be carried out using the subclade 8b strains isolated in Chiba Prefecture during 1996–2014 and the strains isolated in other areas of Japan in the 1990s.

## Supporting information

S1 Fig Location of Chiba Prefecture in Japan. Red area indicates Chiba Prefecture, Japan.
(PDF)

S2 Fig MST of O157 strains used to construct the SNP set for classification of clade 8 strains. The MST was reconstructed from IS*629* insertion distribution data for O157 strains in clade 8. Black nodes indicate clade 8 strains analyzed by WGS.
(PDF)

S3 Fig MSTs of selected O157 strains in each clade. MSTs were reconstructed from IS*629* insertion distribution data for O157 strains in clade 1, clade 2, clade 3, descendant and ancestral clade 4/5, clade 6, clade 7, clade 9 and clade 12. Colored nodes indicate the strains selected for Stx2 production assays. The colors indicate the phage subtype carried by the strains in that figure, as shown in each figure.
(PDF)

S4 Fig Distribution of levels of Stx2 production in each of subclades 8a and 8b strains in the absence (A) and presence (B) of MMC. The x-axis shows the numbers of strains in subclades 8a and 8b. The y-axis shows the log_2_[Stx2] produced by strains in subclades 8a and 8b. Red and blue blocks indicate subclades 8a and 8b, respectively. Red arrows indicate two subclade 8a strains which did not producing significant levels of Stx2 in the absence and presence of MMC.
(PDF)

S5 Fig Comparison of Stx2 production in strains in subclades 8a and 8b determined in this study and by Ogura et al. [20]. The x-axis shows the log_2_[Stx2] produced by strains in subclades 8a and 8b strains and measured in this study. The y-axis shows the log_2_[Stx2] produced and measured by the method of Ogura et al. [20]. Filled red and blue circles indicate MMC-treated O157 strains in subclades 8a and 8b, respectively. Unfilled red and blue circles indicate MMC-untreated O157 strains in subclades 8a and 8b, respectively.
(PDF)

S1 Table Differences between the O157 clade classifications of Manning et al. [8] and Hirai et al. [10]. (XLSX)

S2 Table Distribution of Stx2 subtypes and Stx2a phage subtypes among O157 strains analyzed by Ogura et al. [20]. (XLSX)

S3 Table Analysis of SNPs at 32 loci used by Griffing et al. [28] for 6 clade 8 strains. (XLSX)

S4 Table Primers used for ARMS-PCR in this study. (XLSX)

S5 Table Primers used for Sanger DNA sequencing in this study. (XLSX)

S6 Table Distribution of subclades and clades of O157 strains isolated from patients with each symptom. (XLSX)

S7 Table Distribution of clades of all epidemiologically unlinked O157 strains. (XLSX)

S8 Table O157 strains in clade 8 that were analyzed in this study and their data summary. (XLSX)

S9 Table O157 strains in other clades that were analyzed in this study and their data summary. (XLSX)

## References

[1] van Soolingen D, Qian L, de Haas PE, Douglas JT, Traore H, Portaels F, et al Predominance of a single genotype of *Mycobacterium tuberculosis* in countries of east Asia. Clin Microbiol. 1995; 33 (12): 3234–3238. 10.1128/jcm.33.12.3234-3238.1995 PMC228679 8586708

[2] Leopold SR, Magrini V, Holt NJ, Shaikh N, Mardis ER, Cagno J, et al A precise reconstruction of the emergence and constrained radiations of *Escherichia coli* O157 portrayed by backbone concatenomic analysis. Proc Natl Acad Sci USA. 2009; 106 (21), 8713–8718. doi: 10.1073/pnas.0812949106 19439656 10.1073/pnas.0812949106 PMC2689004

[3] Monday SR, Whittam TS, Feng PC. Genetic and evolutionary analysis of mutations in the *gusA* gene that cause the absence of beta-glucuronidase activity in *Escherichia coli* O157:H7. J Infect Dis. 2001; 184 (7), 918–921. doi: 10.1086/323154 11510000 10.1086/323154

[4] Mestre O, Luo T, Dos Vultos T, Kremer K, Murray A, Namouchi A, et al, Phylogeny of *Mycobacterium tuberculosis* Beijing strains constructed from polymorphisms in genes involved in DNA replication, recombination and repair. PLoS One. 2011; 6 (1), e16020 doi: 10.1371/journal.pone.0016020 21283803 10.1371/journal.pone.0016020 PMC3024326

[5] Shaikh N, Holt NJ, Johnson JR, Tarr PI. Fim operon variation in the emergence of Enterohemorrhagic *Escherichia coli*: An evolutionary and functional analysis. FEMS Microbiol Lett. 2007; 273 (1): 58–63. doi: 10.1111/j.1574-6968.2007.00781.x 17559392 10.1111/j.1574-6968.2007.00781.x

[6] Kim J, Nietfeldt J, Ju J, Wise J, Fegan N, Desmarchelier P, Benson AK. Ancestral divergence, genome diversification, and phylogeographic variation in subpopulations of sorbitol-negative, beta-glucuronidase-negative enterohemorrhagic *Escherichia coli* O157. J Bacteriol. 2001; 183 (23): 6885–6897. doi: 10.1128/JB.183.23.6885-6897.2001 11698378 10.1128/JB.183.23.6885-6897.2001 PMC95530

[7] Shaikh N, Tarr PI. *Escherichia coli* O157:H7 Shiga toxin-encoding bacteriophages: integrations, excisions, truncations, and evolutionary implications. J Bacteriol. 2003; 185 (12): 3596–3605. doi: 10.1128/JB.185.12.3596-3605.2003 12775697 10.1128/JB.185.12.3596-3605.2003 PMC156235

[8] Manning SD, Motiwala AS, Springman AC, Qi W, Lacher DW, Ouellette LM, et al Variation in virulence among clades of *Escherichia coli* O157:H7 associated with disease outbreaks. Proc Natl Acad Sci U S A. 2008 (12); 105: 4868–4873. doi: 10.1073/pnas.0710834105 18332430 10.1073/pnas.0710834105 PMC2290780

[9] Yokoyama E, Hirai S, Hashimoto R, Uchimura M. Clade analysis of enterohemorrhagic *Escherichia coli* serotype O157:H7/H- strains and hierarchy of their phylogenetic relationships. Infect Genet Evol. 2012; 12 (8), 1724–1728. doi: 10.1016/j.meegid.2012.07.003 22846398 10.1016/j.meegid.2012.07.003

[10] Hirai S, Yokoyama E, Yamamoto T. Linkage disequilibrium of the IS*629* insertion among different clades of enterohemorrhagic *Escherichia coli* O157:H7/H-strains. Infect Genet Evol. 2013; 18, 94–99. doi: 10.1016/j.meegid.2013.05.006 23684793 10.1016/j.meegid.2013.05.006

[11] Iyoda S, Manning SD, Seto K, Kimata K, Isobe J, Etoh Y, et al Phylogenetic Clades 6 and 8 of Enterohemorrhagic *Escherichia coli* O157:H7 With Particular stx Subtypes are More Frequently Found in Isolates From Hemolytic Uremic Syndrome Patients Than From Asymptomatic Carriers. Open Forum Infect Dis. 2014; 1(2): ofu061 doi: 10.1093/ofid/ofu061 25734131 10.1093/ofid/ofu061 PMC4281788

[12] Melton-Celsa A, Mohawk K, Teel L, O’Brien A. Pathogenesis of Shiga-toxin producing *Escherichia coli*. Curr Top Microbiol Immunol. 2012; 357: 67–103. doi: 10.1007/82_2011_176 21915773 10.1007/82_2011_176

[13] Scheutz F, Teel LD, Beutin L, Piérard D, Buvens G, Karch H, et al Multicenter evaluation of a sequence-based protocol for subtyping Shiga toxins and standardizing Stx nomenclature. J Clin Microbiol. 2012; 50 (9): 2951–2963. doi: 10.1128/JCM.00860-12 22760050 10.1128/JCM.00860-12 PMC3421821

[14] Friedrich AW, Bielaszewska M, Zhang WL, Pulz M, Kuczius T, Ammon A, et al *Escherichia coli* harboring Shiga toxin 2 gene variants: frequency and association with clinical symptoms. J Infect Dis; 2002: 185(1):74–84. doi: 10.1086/338115 11756984 10.1086/338115

[15] Persson S, Olsen KE, Ethelberg S, Scheutz F. Subtyping method for *Escherichia coli* shiga toxin (verocytotoxin) 2 variants and correlations to clinical manifestations. J Clin Microbiol. 2007; 45 (6): 2020–2024. doi: 10.1128/JCM.02591-06 17446326 10.1128/JCM.02591-06 PMC1933035

[16] Abu-Ali GS, Ouellette LM, Henderson ST, Lacher DW, Riordan JT, Whittam TS, et al Increased adherence and expression of virulence genes in a lineage of *Escherichia coli* O157:H7 commonly associated with human infections. PLoS One. 2010; 5 (4): e10167 doi: 10.1371/journal.pone.0010167 20422047 10.1371/journal.pone.0010167 PMC2858043

[17] Neupane M, Abu-Ali GS, Mitra A, Lacher DW, Manning SD, Riordan JT. Shiga toxin 2 overexpression in *Escherichia coli* O157:H7 strains associated with severe human disease. Microb Pathog. 2011; 51 (6): 466–470. doi: 10.1016/j.micpath.2011.07.009 21864671 10.1016/j.micpath.2011.07.009 PMC3205445

[18] Hirai S, Yokoyama E, Etoh Y, Seto J, Ichihara S, Suzuki Y, et al Putative classification of clades of enterohemorrhagic *Escherichia coli* O157 using an IS-printing system. Lett Appl Microbiol. 2015; 61 (3): 267–273. doi: 10.1111/lam.12448 26031479 10.1111/lam.12448

[19] Kusumoto M, Ooka T, Nishiya Y, Ogura Y, Saito T, Sekine Y, et al Insertion sequence-excision enhancer removes transposable elements from bacterial genomes and induces various genomic deletions. Nat Commun. 2011; 2: 152 doi: 10.1038/ncomms1152 21224843 10.1038/ncomms1152

[20] Ogura Y, Mondal SI, Islam MR, Mako T, Arisawa K, Katsura K, et al The Shiga toxin 2 production level in enterohemorrhagic *Escherichia coli* O157:H7 is correlated with the subtypes of toxin-encoding phage. Sci Rep. 2015; 5:16663 doi: 10.1038/srep16663 26567959 10.1038/srep16663 PMC4645166

[21] Yokoyama E, Etoh Y, Ichihara S, Horikawa K, Konishi N, Kai A, et al Emergence of enterohemorrhagic *Escherichia coli* serovar O157 strains in clade 8 with highly similar pulsed-field gel electrophoresis patterns. J Food Prot. 2011; 74 (8): 1324–1327. doi: 10.4315/0362-028X.JFP-10-461 21819660 10.4315/0362-028X.JFP-10-461

[22] Delannoy S, Mariani-Kurkdjian P, Bonacorsi S, Liguori S, Fach P. Characteristics of emerging human-pathogenic *Escherichia coli* O26:H11 strains isolated in France between 2010 and 2013 and carrying the *stx2d* gene only. J Clin Microbiol. 2015; 53 (2): 486–492. doi: 10.1128/JCM.02290-14 25428148 10.1128/JCM.02290-14 PMC4298503

[23] Etoh Y, Hirai S, Ichihara S, Maeda E, Yokoyama E, Sera N, et al Evolutionary model of the divergence of enterohemorrhagic *Escherichia coli* O157 lineage I/II clades reconstructed from high resolution melting and Shiga-like toxin 2 analyses. Infect Genet Evol. 2014; 24: 140–145. doi: 10.1016/j.meegid.2014.03.013 24667048 10.1016/j.meegid.2014.03.013

[24] Hirai S, Yokoyama E, Etoh Y, Seto J, Ichihara S, Suzuki Y, et al Analysis of the population genetics of clades of enterohaemorrhagic *Escherichia coli* O157:H7/H- isolated in three areas in Japan. J Appl Microbiol. 2014; 117 (4): 1191–1197. doi: 10.1111/jam.12604 25047966 10.1111/jam.12604

[25] Yang Z, Kovar J, Kim J, Nietfeldt J, Smith DR, Moxley RA, et al Identification of common subpopulations of non-sorbitol-fermenting, beta-glucuronidase-negative *Escherichia coli* O157:H7 from bovine production environments and human clinical samples. Appl Environ Microbiol. 2004; 70 (11): 6846–6854. doi: 10.1128/AEM.70.11.6846-6854.2004 15528552 10.1128/AEM.70.11.6846-6854.2004 PMC525184

[26] Ooka T, Terajima J, Kusumoto M, Iguchi A, Kurokawa K, Ogura Y, et al Development of a multiplex PCR-based rapid typing method for enterohemorrhagic *Escherichia coli* O157 strains. J Clin Microbiol. 2009; 47 (9): 2888–2894. doi: 10.1128/JCM.00792-09 19641072 10.1128/JCM.00792-09 PMC2738077

[27] Wang G, Clark CG, Rodgers FG. Detection in *Escherichia coli* of genes encoding the major virulence factors, the genes defining of the O157:H7 serotype, and components of the type 2 Shiga toxin family by multiplex PCR. J. Clin. Microbiol. 2002; 40 (10): 3613–3619. doi: 10.1128/JCM.40.10.3613-3619.2002 12354854 10.1128/JCM.40.10.3613-3619.2002 PMC130888

[28] Griffing SM, MacCannell DR, Schmidtke AJ, Freeman MM, Hyytiä-Trees E, Gerner-Smidt P, et al Canonical Single Nucleotide Polymorphisms (SNPs) for High-Resolution Subtyping of Shiga-Toxin Producing *Escherichia coli* (STEC) O157:H7. PLoS One. 2015; 10 (7): e0131967 doi: 10.1371/journal.pone.0131967 26132731 10.1371/journal.pone.0131967 PMC4488506

[29] Yokoyama E, Hirai S, Ishige T, Murakami S. Single-Nucleotide Polymorphisms in the Whole-Genome Sequence Data of Shiga Toxin-Producing *Escherichia coli* O157:H7/H- Strains by Cultivation. Curr Microbiol. 2017; 74 (4): 425–430. doi: 10.1007/s00284-017-1208-z 28197720 10.1007/s00284-017-1208-z

[30] Yokoyama E, Ando N, Ohta T, Kanada A, Shiwa Y, Ishige T, et al A novel subpopulation of *Salmonella enterica* serovar Infantis strains isolated from broiler chicken organs other than the gastrointestinal tract. Vet Microbiol. 2015; 175 (2–4): 312–318. doi: 10.1016/j.vetmic.2014.11.024 25542287 10.1016/j.vetmic.2014.11.024

[31] Newton CR, Graham A, Heptinstall LE, Powell SJ, Summers C, Kalsheker N, et al Analysis of any point mutation in DNA. The amplification refractory mutation system (ARMS). Nucleic Acids Res. 1989; 17 (7): 2503–2516. 2785681 10.1093/nar/17.7.2503 PMC317639

[32] Tamura K, Stecher G, Peterson D, Filipski A, Kumar S. MEGA6: Molecular Evolutionary Genetics Analysis version 6.0. Mol Biol Evol. 2013; 30 (12): 2725–2729. doi: 10.1093/molbev/mst197 24132122 10.1093/molbev/mst197 PMC3840312

[33] Peakall R, Smouse PE. GenAlEx 6.5: genetic analysis in Excel. Population genetic software for teaching and research—an update. Bioinformatics. 2012; 28 (19): 2537–2539. doi: 10.1093/bioinformatics/bts460 22820204 10.1093/bioinformatics/bts460 PMC3463245

[34] Gaston MA, Hunter PR. Efficient selection of tests for bacteriological typing schemes. J Clin Pathol. 1989; 42 (7): 763–766. 2668344 10.1136/jcp.42.7.763 PMC1142031

[35] Izumiya H, Pei Y, Terajima J, Ohnishi M, Hayashi T, Iyoda S, et al New system for multilocus variable-number tandem-repeat analysis of the enterohemorrhagic *Escherichia coli* strains belonging to three major serogroups: O157, O26, and O111. Microbiol Immunol. 2010; 54 (10): 569–577. doi: 10.1111/j.1348-0421.2010.00252.x 21118294 10.1111/j.1348-0421.2010.00252.x

[36] Wagner PL, Neely MN, Zhang X, Acheson DW, Waldor MK, Friedman DI. Role for a phage promoter in Shiga toxin 2 expression from a pathogenic *Escherichia coli* strain. J Bacteriol. 2001;183 (6): 2081–2085. doi: 10.1128/JB.183.6.2081-2085.2001 11222608 10.1128/JB.183.6.2081-2085.2001 PMC95105

[37] Ichimura K, Shimizu T, Matsumoto A, Hirai S, Yokoyama E, Takeuchi H, et al Nitric oxide-enhanced Shiga toxin production was regulated by Fur and RecA in enterohemorrhagic *Escherichia coli* O157. Microbiologyopen. Forth coming 2017. 10.1002/mbo3.461 PMC5552940 28294553

[38] Shimizu T, Hirai S, Yokoyama E, Ichimura K, Noda M. An evolutionary analysis of nitric oxide reductase gene *norV* in enterohemorrhagic *Escherichia coli* O157. Infect Genet Evol. 2015; 33: 176–181. doi: 10.1016/j.meegid.2015.04.027 25936496 10.1016/j.meegid.2015.04.027

[39] Yin S, Rusconi B, Sanjar F, Goswami K, Xiaoli L, Eppinger M, et al *Escherichia coli* O157:H7 strains harbor at least three distinct sequence types of Shiga toxin 2a-converting phages. BMC Genomics. 2015; 16: 733 doi: 10.1186/s12864-015-1934-1 26416807 10.1186/s12864-015-1934-1 PMC4587872

[40] Serra-Moreno R, Jofre J, Muniesa M. The CI repressors of Shiga toxin-converting prophages are involved in coinfection of *Escherichia coli* strains, which causes a down regulation in the production of Shiga toxin 2. J Bacteriol. 2008; 190 (13): 4722–4735. doi: 10.1128/JB.00069-08 18469095 10.1128/JB.00069-08 PMC2446792

[41] Muniesa M, de Simon M, Prats G, Ferrer D, Pañella H, Jofre J. Shiga toxin 2-converting bacteriophages associated with clonal variability in *Escherichia coli* O157:H7 strains of human origin isolated from a single outbreak. Infect Immun. 2003; 71 (8): 4554–4562. doi: 10.1128/IAI.71.8.4554-4562.2003 12874335 10.1128/IAI.71.8.4554-4562.2003 PMC166033

[42] Amigo N, Mercado E, Bentancor A, Singh P, Vilte D, Gerhardt E, et al Clade 8 and clade 6 strains of *Escherichia coli* O157:H7 from cattle in Argentina have hypervirulent-like phenotypes. PLoS One. 2015; 10 (6): e0127710 doi: 10.1371/journal.pone.0127710 26030198 10.1371/journal.pone.0127710 PMC4452545

[43] Amigo N, Zhang Q, Amadio A, Zhang Q, Silva WM, Cui B, et al Overexpressed proteins in hypervirulent clade 8 and clade 6 strains of *Escherichia coli* O157:H7 compared to *E*. *coli* O157:H7 EDL933 clade 3 strain. PLoS One. 2016; 11 (11): e0166883 doi: 10.1371/journal.pone.0166883 27880834 10.1371/journal.pone.0166883 PMC5120812

