# Correction: Enterohemorrhagic *Escherichia coli* O157 subclade 8b strains in Chiba Prefecture, Japan, produced larger amounts of Shiga toxin 2 than strains in subclade 8a and other clades

**DOI:** 10.1371/journal.pone.0196027

**Published:** 2018-04-16

**Authors:** Shinichiro Hirai, Eiji Yokoyama, Taku Wakui, Taichiro Ishige, Masaki Nakamura

There is an error in the seventh sentence of the Abstract. The correct sentence is: On the other hand, a recent study reported that the Stx2 production level in O157 strains was determined mainly by the subtypes of Stx2a phage (ϕStx2a_α, β, γ, δ, ε, and ζ).

There are errors in the penultimate and final sentences of the Abstract. The correct sentences are: In the presence of MMC, subclade 8b strains produced significantly greater amounts of Stx2 than clade 6 strains carrying ϕStx2a_δ. In this study, we propose that Stx2 production in subclade 8b strains in the presence of MMC might be enhanced due to genetic factors other than ϕStx2a_δ.

There is an error in the third sentence of the final paragraph of the Discussion section. The correct sentence is: The level of Stx2 production in subclade 8b strains isolated in Chiba Prefecture in the presence of MMC may be enhanced by genetic factors other than ϕStx2a_δ.
