# Supplementary figures and images for: Enterohemorrhagic Escherichia coli O157 subclade 8b strains in Chiba Prefecture, Japan, produced larger amounts of Shiga toxin 2 than strains in subclade 8a and other clades
Source: PLoS One. 2018 Jan 30;13(1):e0191834. doi: 10.1371/journal.pone.0191834 (PMC5790261; doi:10.1371/journal.pone.0191834)

S1 Fig

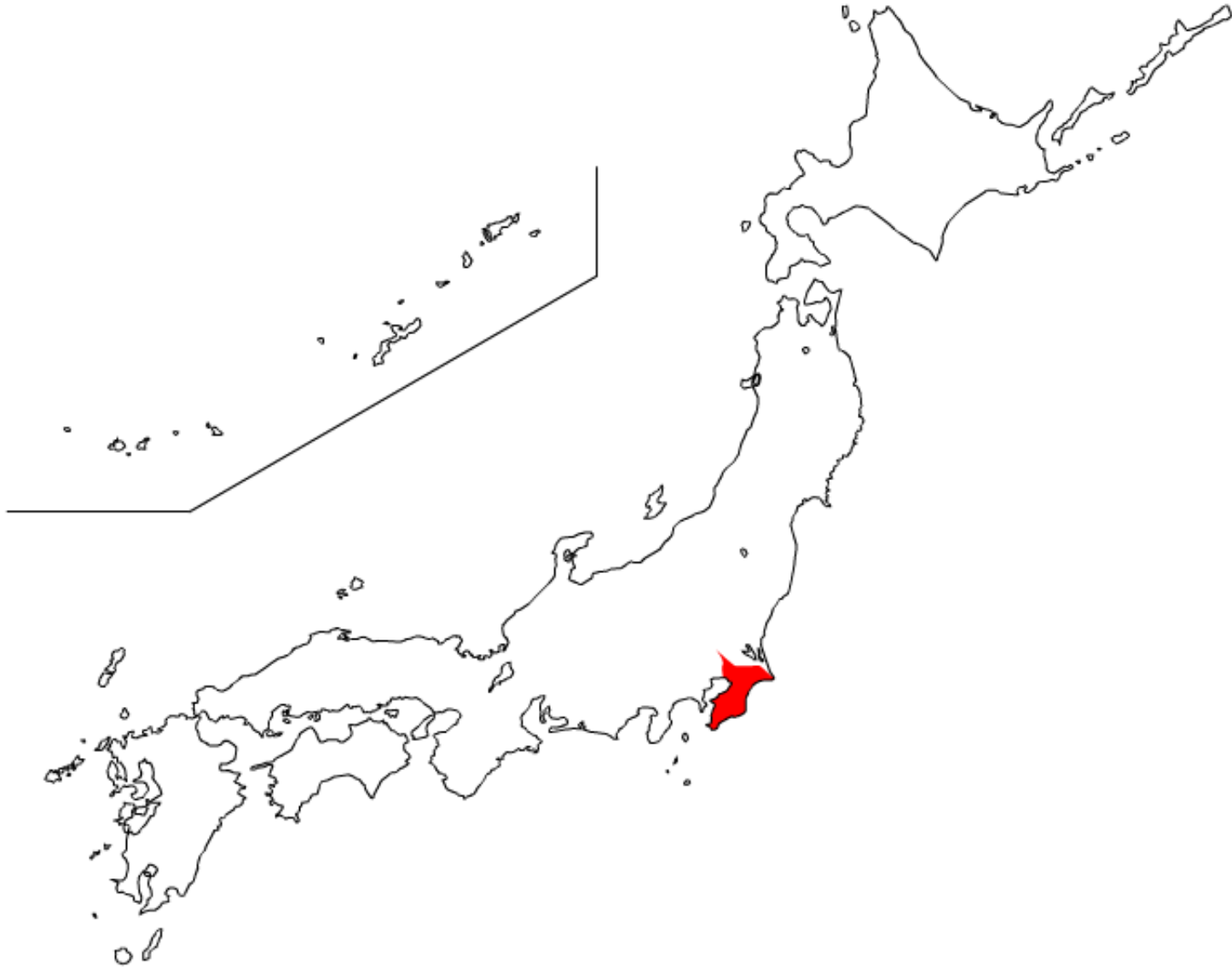

Supplement: S1 Fig — Red area indicates Chiba Prefecture, Japan. (PDF) [file pone.0191834.s001.pdf]

S2 Fig

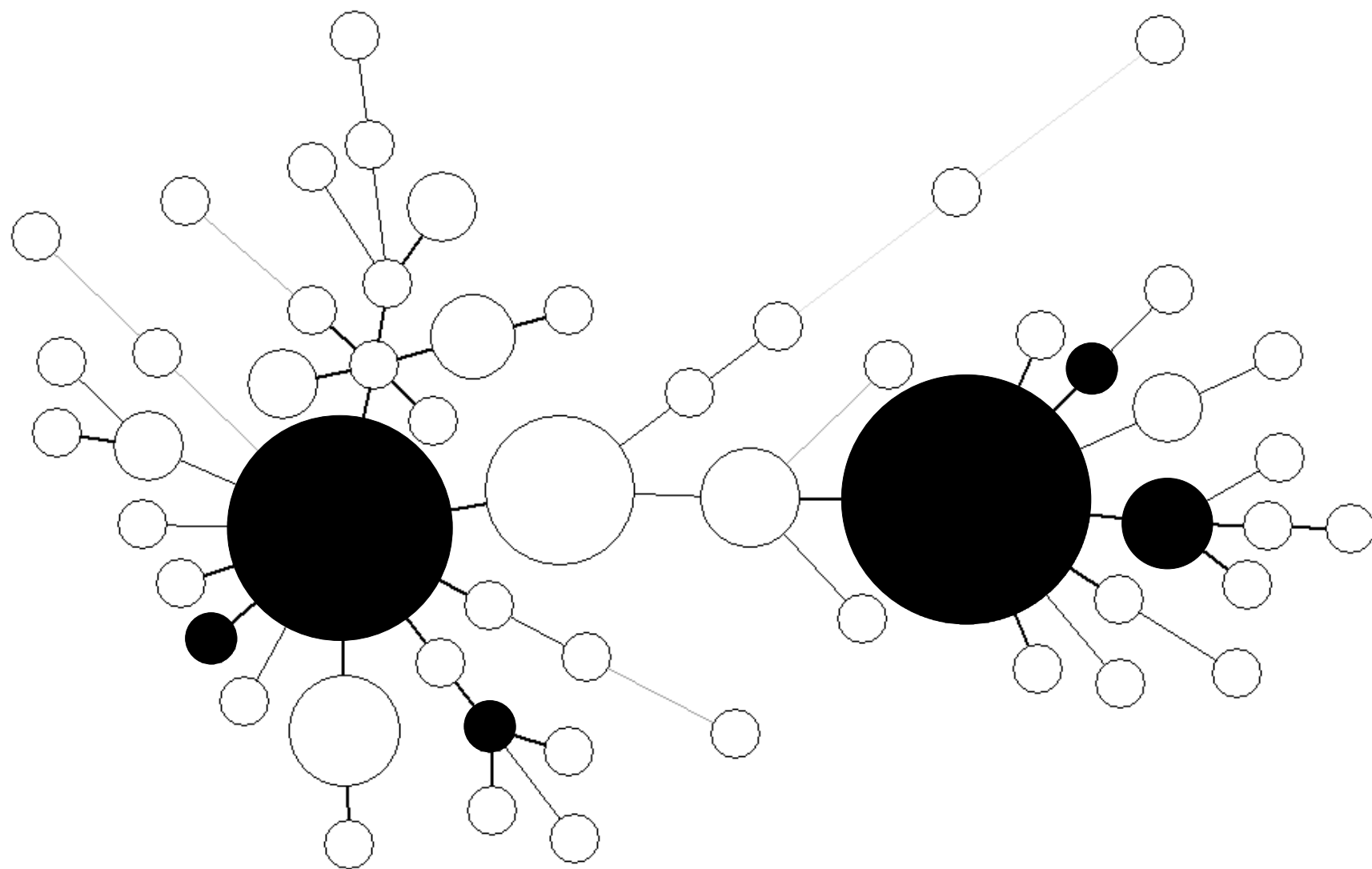

Supplement: S2 Fig — The MST was reconstructed from IS629 insertion distribution data for O157 strains in clade 8. Black nodes indicate clade 8 strains analyzed by WGS. (PDF) [file pone.0191834.s002.pdf]

(A) MMC -

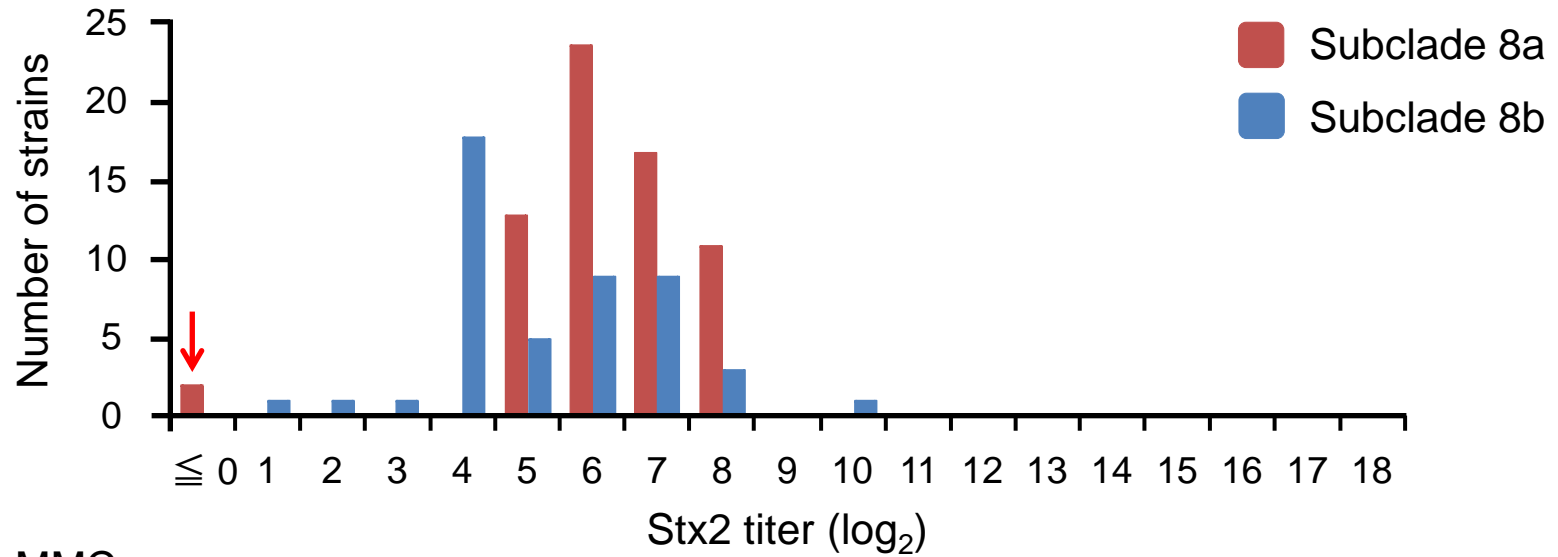

(B) MMC +

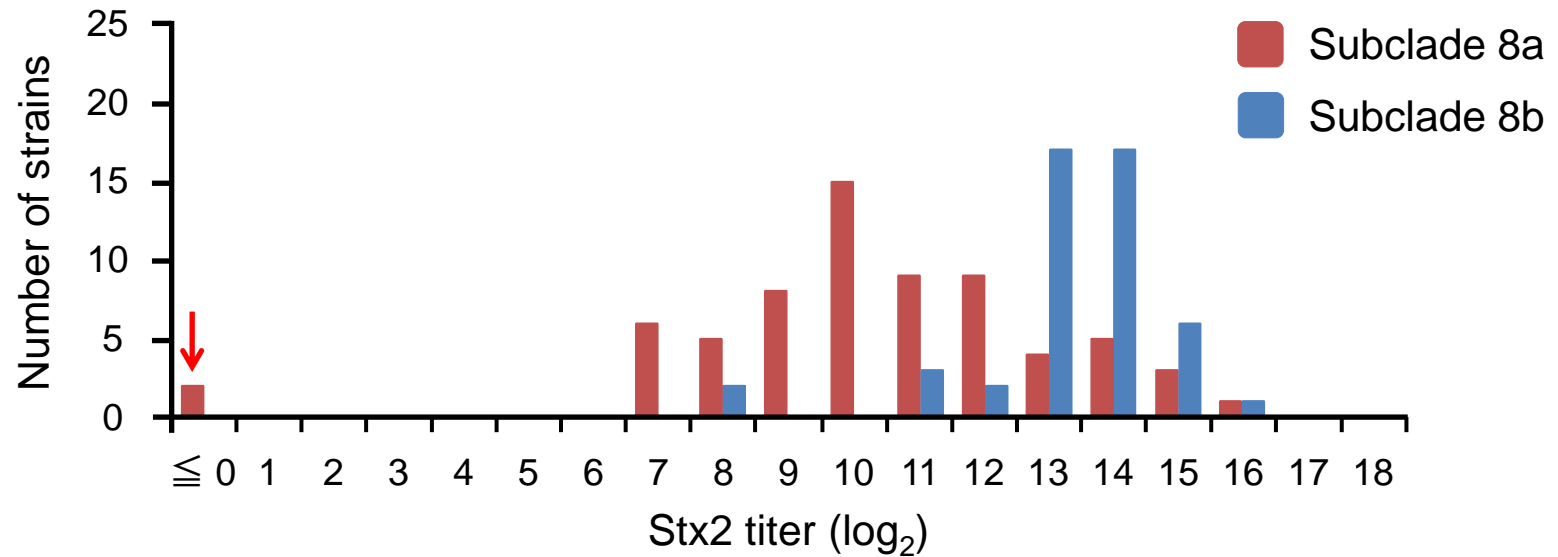

Supplement: S4 Fig — The x-axis shows the numbers of strains in subclades 8a and 8b. The y-axis shows the log2[Stx2] produced by strains in subclades 8a and 8b. Red and blue blocks indicate subclades 8a and 8b, respectively. Red arrows indicate two subclade 8a strains which did not producing significant levels of Stx2 in the absence and presence of MMC. (PDF) [file pone.0191834.s004.pdf]

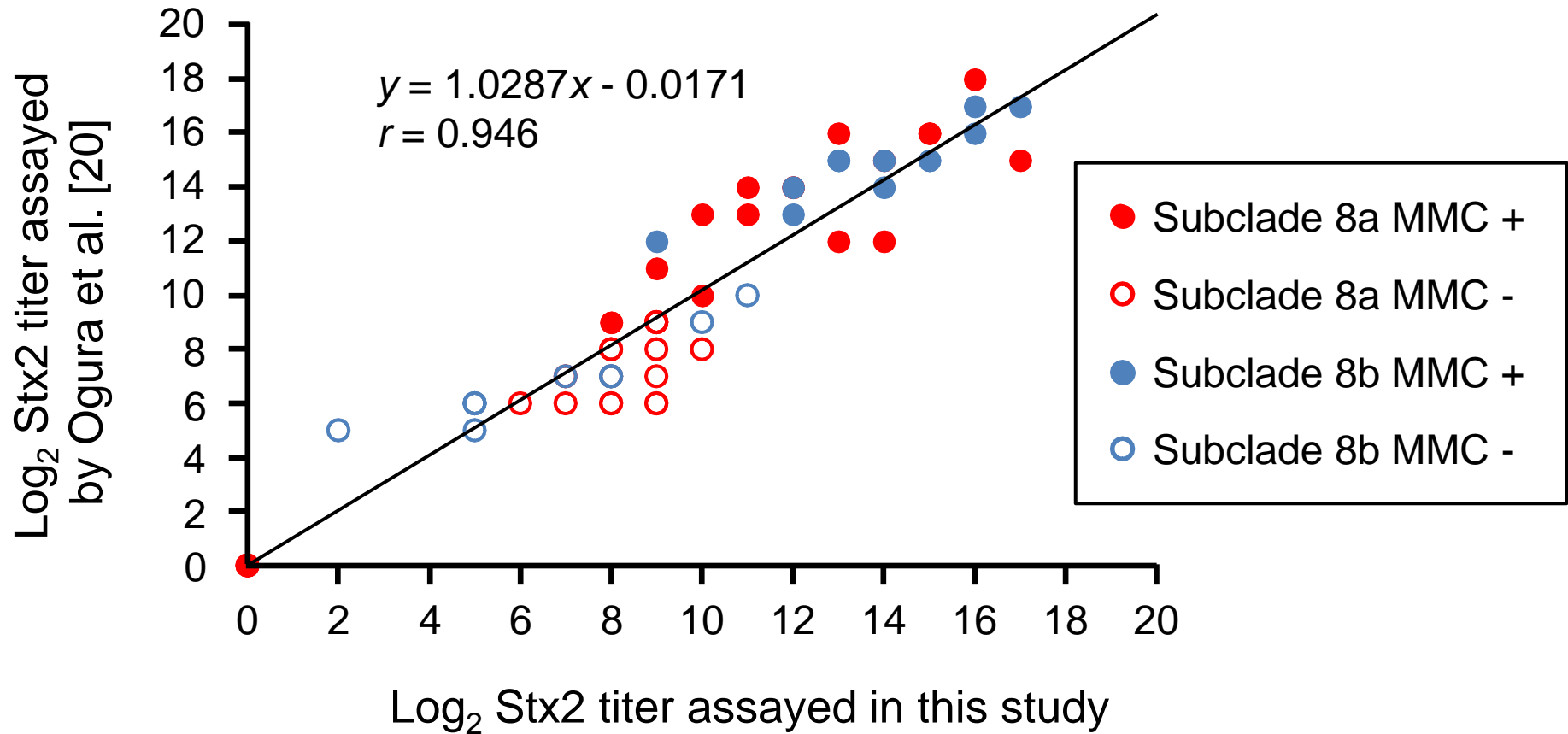

Supplement: S5 Fig — The x-axis shows the log2[Stx2] produced by strains in subclades 8a and 8b strains and measured in this study. The y-axis shows the log2[Stx2] produced and measured by the method of Ogura et al. [20]. Filled red and blue circles indicate MMC-treated O157 strains in subclades 8a and 8b, respectively. Unfilled red and blue circles indicate MMC-untreated O157 strains in subclades 8a and 8b, respectively. (PDF) [file pone.0191834.s005.pdf]
