# Supplementary material for: Enterohemorrhagic Escherichia coli O157 subclade 8b strains in Chiba Prefecture, Japan, produced larger amounts of Shiga toxin 2 than strains in subclade 8a and other clades
Source: PLoS One. 2018 Jan 30;13(1):e0191834. doi: 10.1371/journal.pone.0191834 (PMC5790261; doi:10.1371/journal.pone.0191834)

S3 Fig

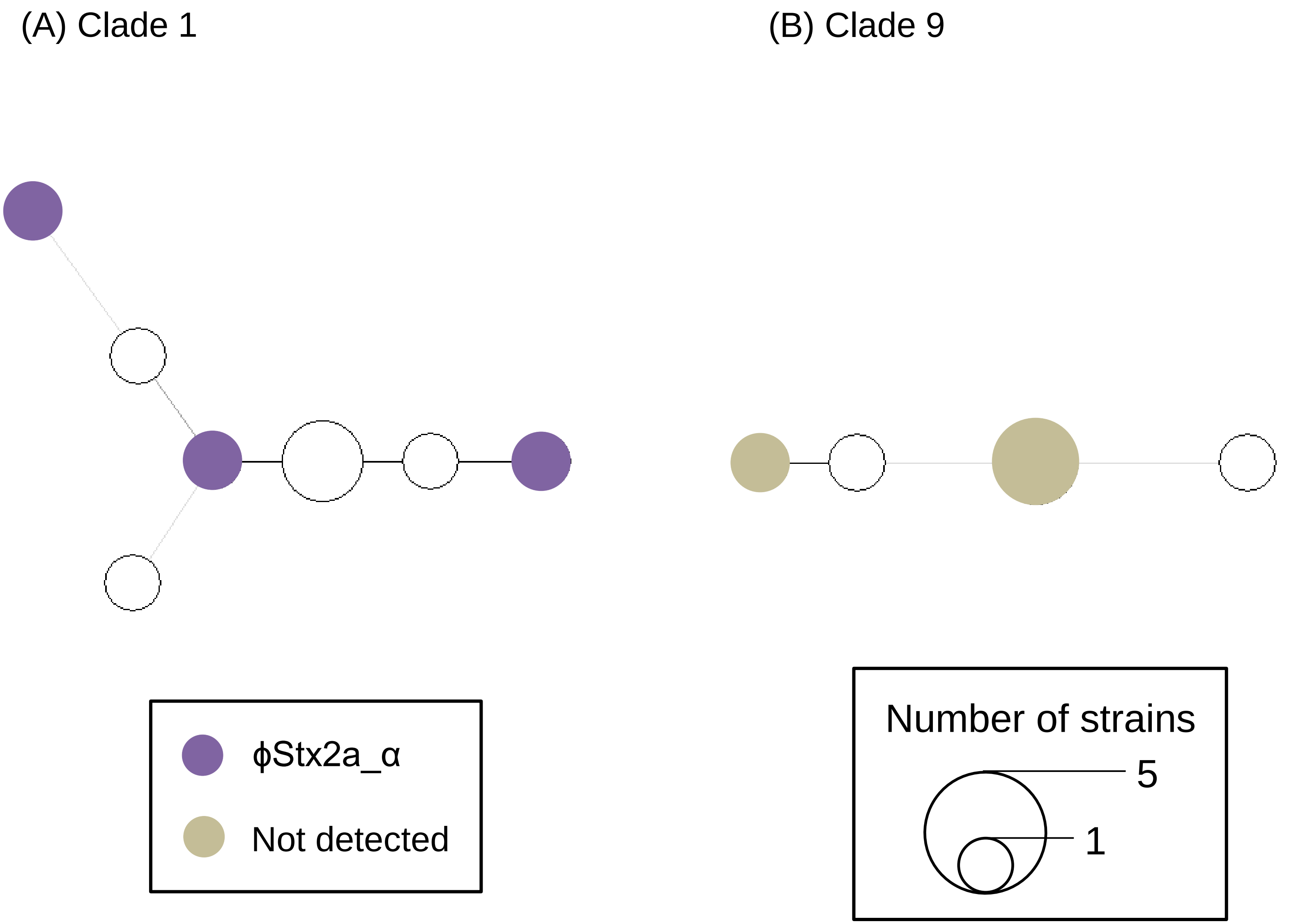

(C) Clade 2

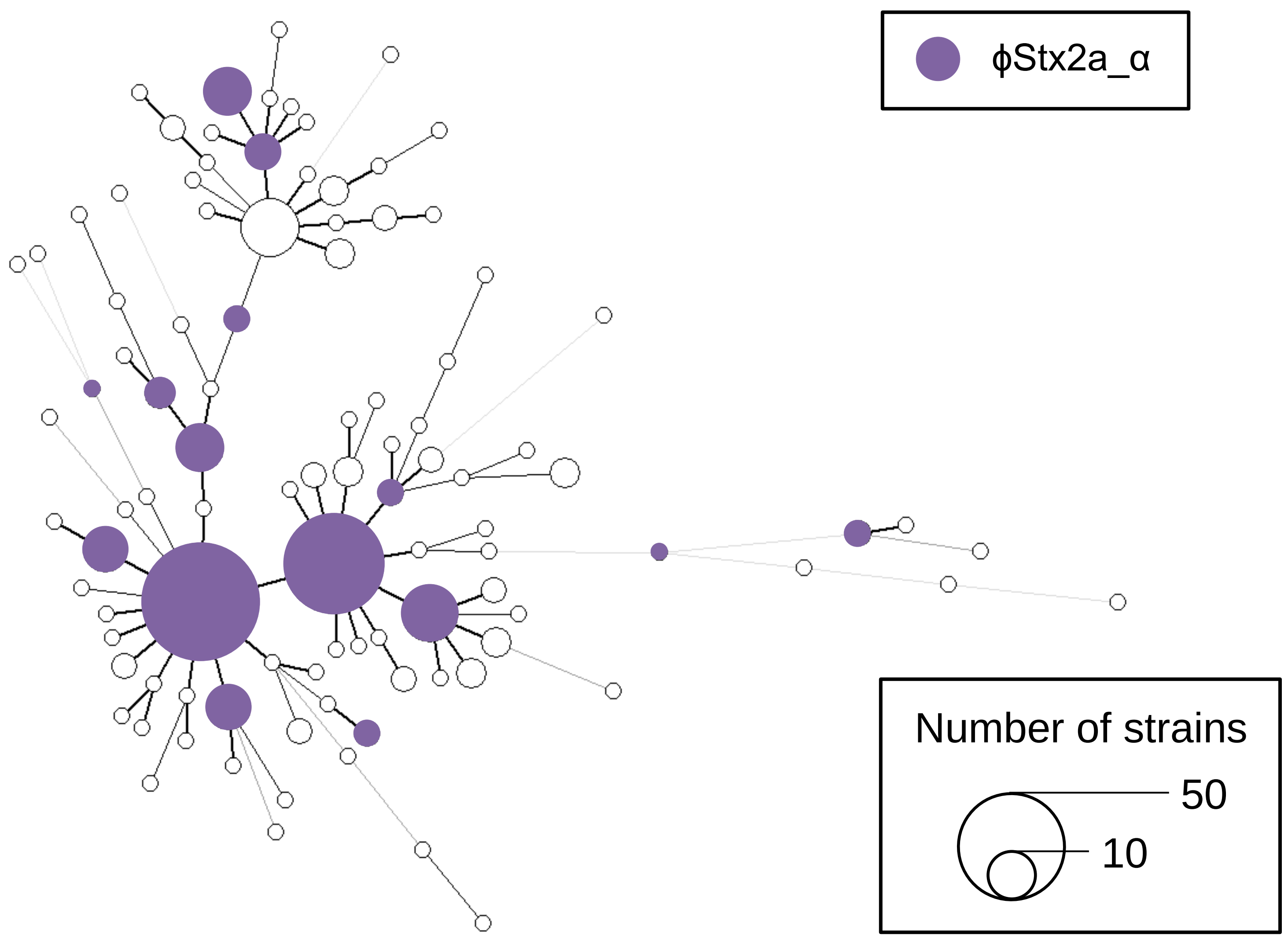

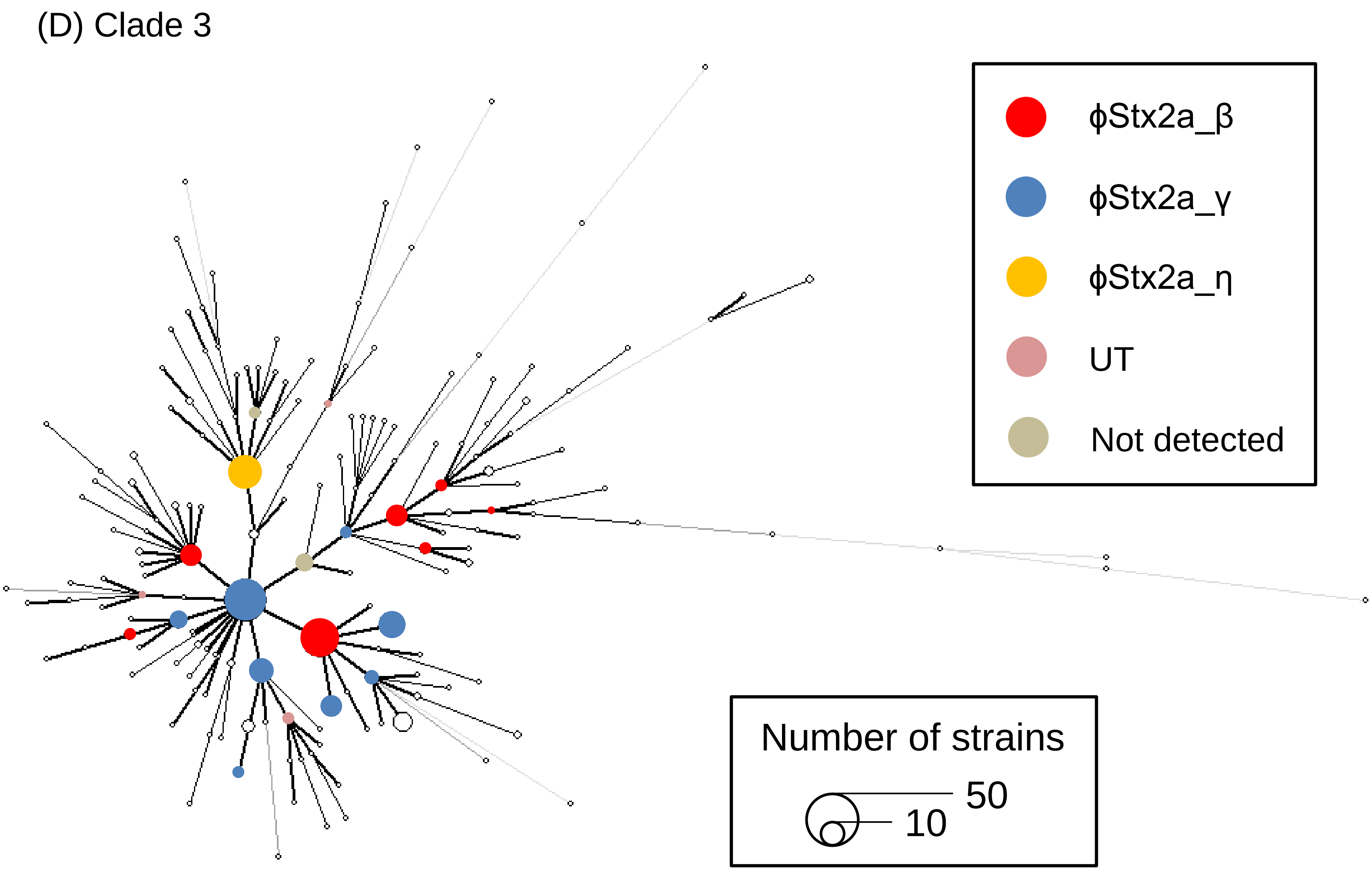
(D) Clade 3
ϕStx2a_β
ϕStx2a_γ
ϕStx2a_η
UT
Not detected
Number of strains
50
10

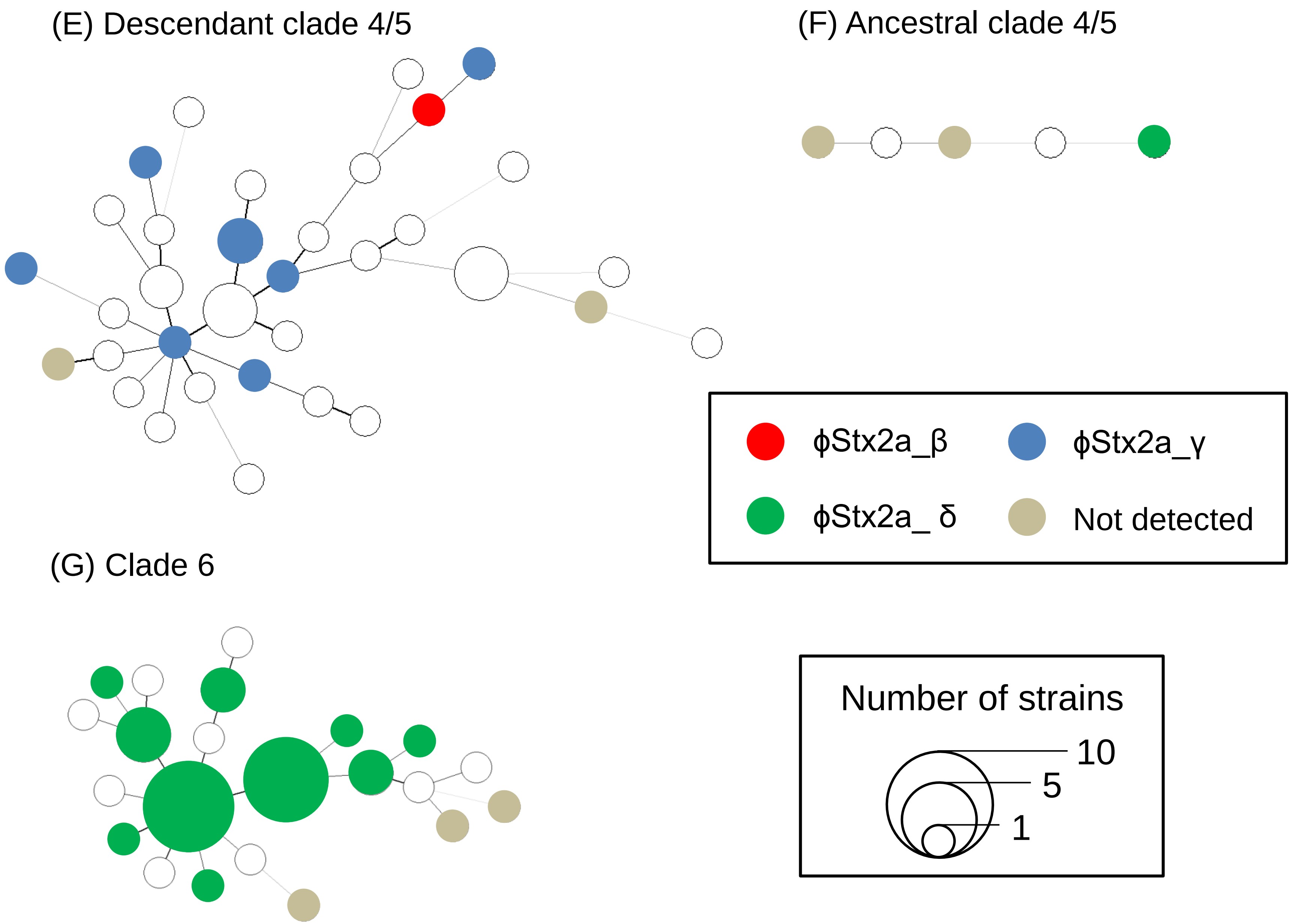
(E) Descendant clade 4/5
(F) Ancestral clade 4/5
(G) Clade 6
ϕStx2a_β
ϕStx2a_γ
ϕStx2a_ δ
Not detected
Number of strains
10
5
1

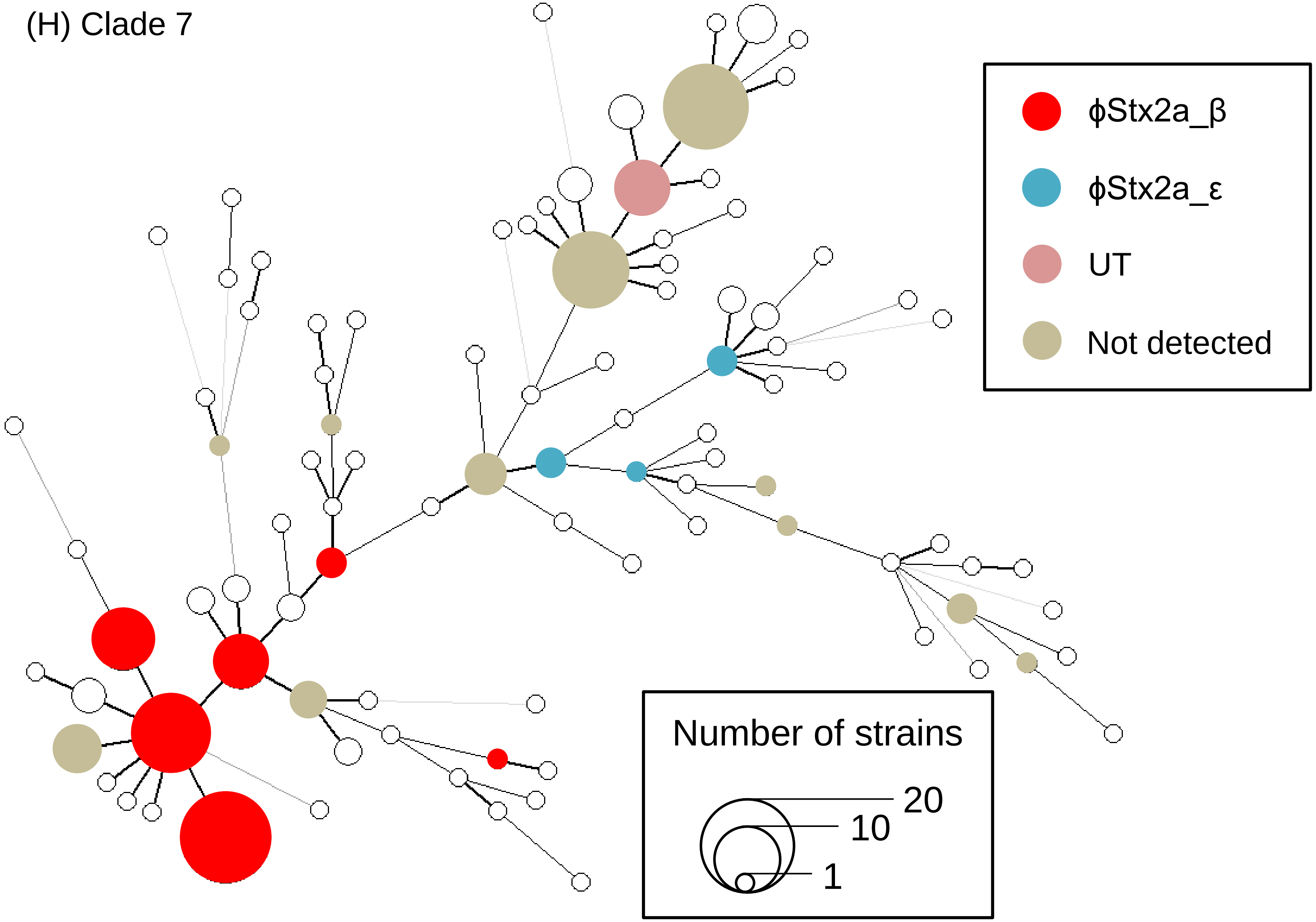

(H) Clade 7
ϕStx2a_β
ϕStx2a_ε
UT
Not detected
Number of strains
20
10
1

(I) Clade 12

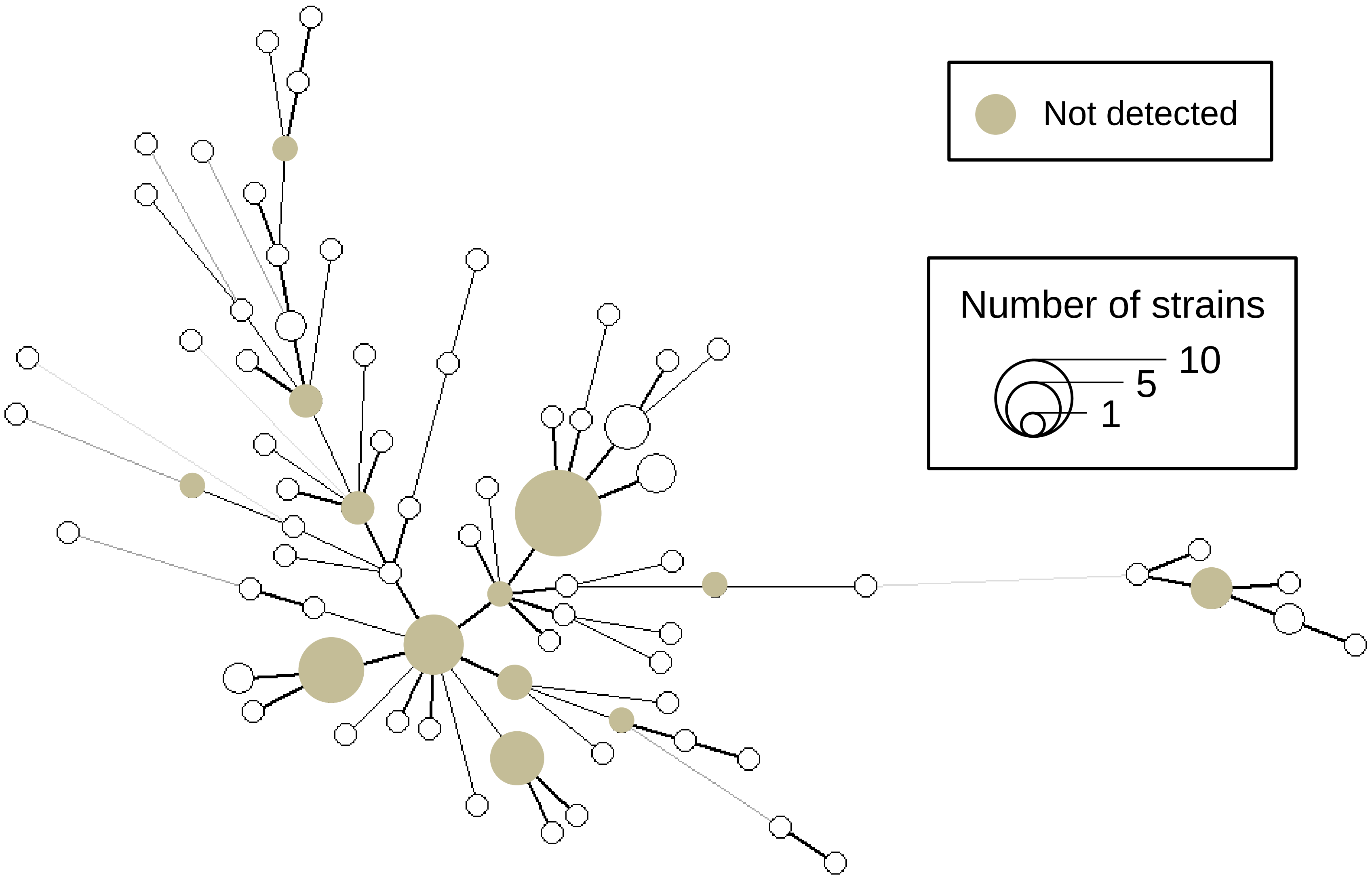

Supplement: S3 Fig — MSTs were reconstructed from IS629 insertion distribution data for O157 strains in clade 1, clade 2, clade 3, descendant and ancestral clade 4/5, clade 6, clade 7, clade 9 and clade 12. Colored nodes indicate the strains selected for Stx2 production assays. The colors indicate the phage subtype carried by the strains in that figure, as shown in each figure. (PDF) [file pone.0191834.s003.pdf]
